# The bacterial sulfur cycle in expanding dysoxic and euxinic marine waters

**DOI:** 10.1111/1462-2920.15265

**Published:** 2020-10-18

**Authors:** Daan M. van Vliet, F.A. Bastiaan von Meijenfeldt, Bas E. Dutilh, Laura Villanueva, Jaap S. Sinninghe Damsté, Alfons J.M. Stams, Irene Sánchez‐Andrea

**Affiliations:** ^1^ Laboratory of Microbiology Wageningen University and Research, Stippeneng 4, 6708WE Wageningen Netherlands; ^2^ Theoretical Biology and Bioinformatics, Science for Life Utrecht University, Padualaan 8, 3584 CH Utrecht Netherlands; ^3^ Department of Marine Microbiology and Biogeochemistry Royal Netherlands Institute for Sea Research (NIOZ), Utrecht University, Landsdiep 4, 1797 SZ, 't Horntje (Texel) Netherlands; ^4^ Department of Earth Sciences, Faculty of Geosciences Utrecht University, Princetonlaan 8A, 3584 CB Utrecht Netherlands; ^5^ Centre of Biological Engineering University of Minho, Campus de Gualtar, 4710‐057 Braga Portugal

## Abstract

Dysoxic marine waters (DMW, < 1 μM oxygen) are currently expanding in volume in the oceans, which has biogeochemical, ecological and societal consequences on a global scale. In these environments, distinct bacteria drive an active sulfur cycle, which has only recently been recognized for open‐ocean DMW. This review summarizes the current knowledge on these sulfur‐cycling bacteria. Critical bottlenecks and questions for future research are specifically addressed. Sulfate‐reducing bacteria (SRB) are core members of DMW. However, their roles are not entirely clear, and they remain largely uncultured. We found support for their remarkable diversity and taxonomic novelty by mining metagenome‐assembled genomes from the Black Sea as model ecosystem. We highlight recent insights into the metabolism of key sulfur‐oxidizing SUP05 and *Sulfurimonas* bacteria, and discuss the probable involvement of uncultivated SAR324 and BS‐GSO2 bacteria in sulfur oxidation. Uncultivated *Marinimicrobia* bacteria with a presumed organoheterotrophic metabolism are abundant in DMW. Like SRB, they may use specific molybdoenzymes to conserve energy from the oxidation, reduction or disproportionation of sulfur cycle intermediates such as S^0^ and thiosulfate, produced from the oxidation of sulfide. We expect that tailored sampling methods and a renewed focus on cultivation will yield deeper insight into sulfur‐cycling bacteria in DMW.

## Introduction

**Fig 1:**
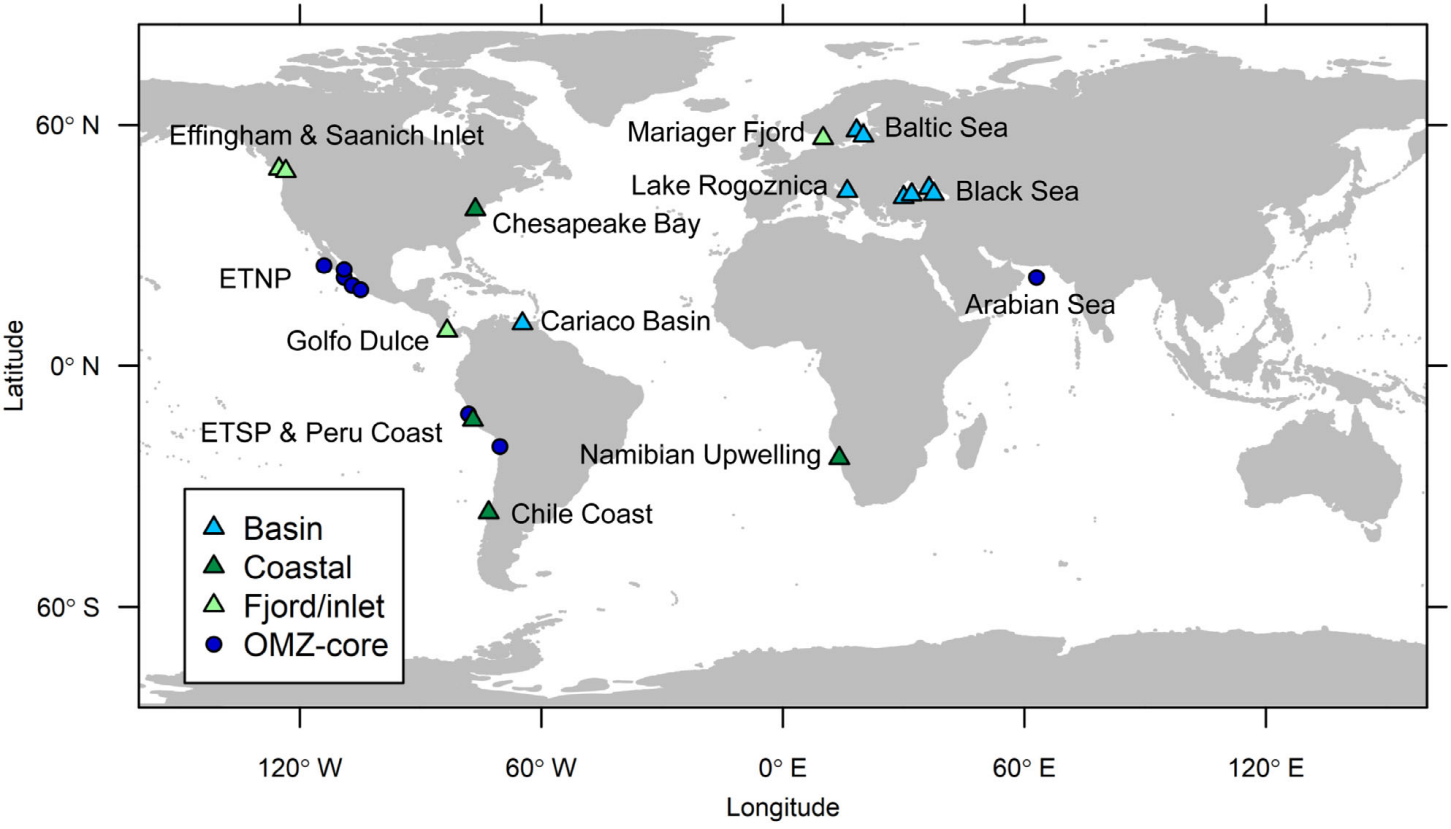
Dysoxic marine waters studied with respect to microorganisms driving the sulfur cycle. Triangles indicate locations that are permanently, seasonally or incidentally euxinic. For a list of studies per location, see Table S1. ETNP, Eastern Tropical North Pacific; ETSP, Eastern Tropical South Pacific; OMZ, oxygen‐minimum zone. [Color figure can be viewed at wileyonlinelibrary.com]

Oxygen deficiency is a rather common phenomenon in marine waters caused by microbial aerobic respiration coupled to the degradation of organic matter, combined with insufficient supply of oxygen through water circulation or diffusion (Canfield *et al*., 2005). Oxygen‐minimum zones (OMZs) are waters in the open ocean containing < 20 μM oxygen occurring between 100 and 1,500 m depth. The largest OMZs are found in the Eastern Tropical North Pacific (ETNP), the Eastern Tropical South Pacific (ETSP) and the Arabian Sea (Fig. 1, Table S1). Together, OMZs amount to 10 million km^3^ or approximately 1% of the ocean's volume (Paulmier and Ruiz‐Pino, 2009). When there is sufficient input of sinking phytoplankton biomass, oxygen concentrations in OMZs can drop to below the common detection level of 1 μM and were considered anoxic and described as ‘anoxic marine zones’ (Ulloa *et al*., 2012). However, using a highly sensitive STOX oxygen sensor, Revsbech and colleagues (2009) and Thamdrup and colleagues (2012) showed that the supposedly anoxic OMZ waters, which will here be termed ‘OMZ core’, still may contain traces of oxygen (< 50 nM). Yet, these sensors have not yet been widely applied in marine sampling campaigns. Coastal waters can similarly experience oxygen deficiency and anoxia, for example in the Namibian Upwelling, Chesapeake Bay and the Pacific South‐American coastal waters (Fig. 1, Table S1).

In enclosed marine basins and fjords, as well as in coastal waters, stratification is a common factor in the development and persistence of oxygen deficiency (Canfield *et al*., 2005). Stratification can even lead to euxinia (Meyer and Kump, 2008), here defined as anoxic conditions with > 0.1 μM sulfide. Several euxinic marine basins, fjords and inlets have been studied with respect to their sulfur cycle and the associated microorganisms (Fig. 1, Table S1). The development of euxinia in OMZs is prevented by a relatively high advection of oxygenated water compared to enclosed environments, and by a negative feedback loop centered around nitrogen loss. Denitrifying and anaerobic ammonia‐oxidizing bacteria in the OMZ convert fixed forms of nitrogen such as ammonium and nitrate into N_2_ at such high rates that OMZs are responsible for 30% to 50% of the total loss of fixed nitrogen from the ocean (Lam and Kuypers, 2011). This causes surface phytoplankton to be limited in nitrogen, which in turn limits the input of organic matter to the OMZs, preventing the depletion of nitrate and nitrite (Canfield, 2006; Boyle *et al*., 2013). Hence, the ensuing development of euxinia is halted, as denitrifying bacteria outcompete sulfate‐reducing ones (Froelich *et al*., 1979; Chen *et al*., 2017).

OMZ core waters, despite being dominated by nitrogen cycling, can also harbor an active sulfur cycle, which has long been overlooked due to the absence of detectable sulfide (Canfield *et al*., 2010; Johnston *et al*., 2014; Carolan *et al*., 2015). Similar conditions are found in some stratified environments where the oxic and euxinic zones are separated by a suboxic zone (Murray *et al*., 1989; Lavik *et al*., 2009; Hawley *et al*., 2014; Findlay *et al*., 2017), here defined to contain no detectable oxygen (< 1 μM) or sulfide (< 0.1 μM) using standard methods. The exact concentration of oxygen in suboxic zones is still unclear. However, the detection of sulfur‐cycling microorganisms suggests an active sulfur cycle in suboxic zones, for instance in the Black Sea and Cariaco Basin (Neretin *et al*., 2007; Rodriguez‐Mora *et al*., 2016). For the purpose of this review, we use the term ‘dysoxic marine water’ (DMW, < 1 μM of oxygen) to describe all marine suboxic zones, OMZ core waters, anoxic waters and euxinic waters (Box 1).

Box 1. **Glossary of definitions for marine oxygen‐deficient environments.**

**Table:** 

Environmental terminology	Definition
Dysoxic water	< 1 μM oxygen
Anoxic water	Not containing oxygen
Euxinic water	Anoxic, > 0.1 μM sulfide
Oxygen‐minimum zone (OMZ)	Open ocean water with < 20 μM oxygen
OMZ core, also known as ‘anoxic marine zone’	Open ocean water with < 50 nM oxygen
Suboxic zone	< 1 μM oxygen, < 0.1 μM sulfide, in between oxic and euxinic zones of stratified waters

Over the last 60 years, DMW has expanded in volume more than fourfold (Schmidtko *et al*., 2017) because of oceanic warming – reducing oxygen solubility – and eutrophication (reviewed by Breitburg *et al*., 2018). This process is expected to continue. In addition, Ulloa and colleagues (2012) have predicted that the deposition of anthropogenically fixed nitrogen will cause OMZ cores to develop euxinia, since it counteracts the nitrogen‐loss‐based negative feedback loop. Potential long‐term, global consequences of expanding marine dysoxia and euxinia include changes in availability of key nutrients (iron, phosphorus, etc.) and trace metals (cadmium, copper, zinc, etc.), and loss of fishery stocks, affecting coastal economies and food security (Breitburg *et al*., 2018). Furthermore, since DMW environments are biogeochemical hotspots for microbial production of the greenhouse gas nitrous oxide (Naqvi *et al*., 2010), their expansion provides a feedback loop that in turn contributes to global warming. In the geological past, the rise of euxinic conditions has led to several mass extinction events such as during the end‐Permian (Meyer and Kump, 2008) and the mid‐Cretaceous (Kamyshny *et al*., 2009).

**Fig 2:**
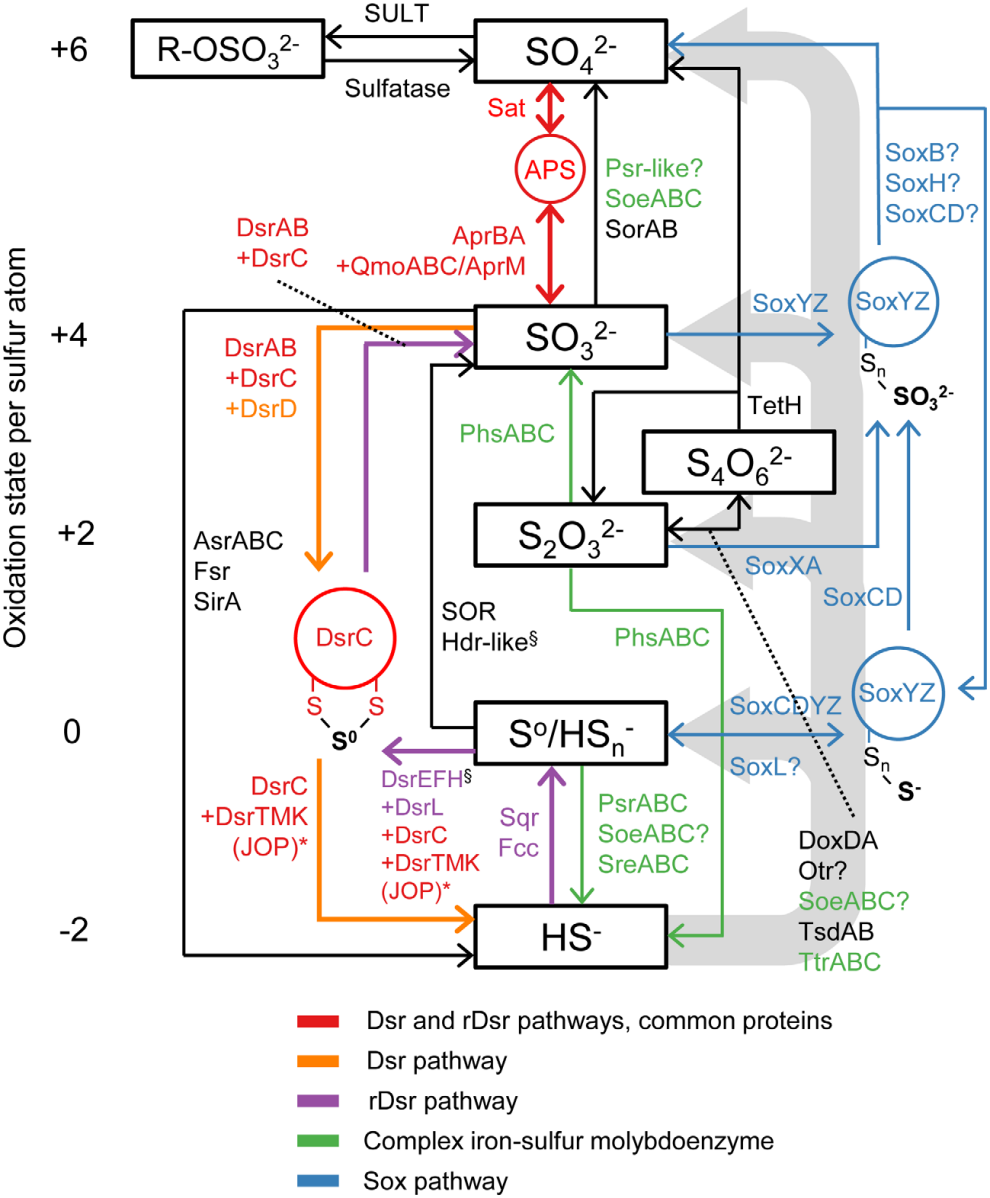
The dissimilatory conversions within the marine sulfur cycle. The oxidation state of the inorganic species is indicated at the left. Abiotic and assimilatory reactions are not indicated, except for the abiotic oxidation of sulfide which is illustrated by wide grey arrows. The S^0^ in DsrC‐trisulfide is considered zero‐valent (Santos *et al*., 2015). The sulfur atom in APS has an oxidation state of +6, and those in tetrathionate have an oxidation state of +2.5. A question mark symbol (?) shows that involvement is uncertain. The asterisk symbol (*) indicates that DsrT is required for sulfide oxidation in green sulfur bacteria (Holkenbrink *et al*., 2011), but is also found in SRB. Protein complexes other than DsrTMK(JOP) can also transfer electrons to DsrC to enable this reaction (Venceslau *et al*., 2014). The section symbol (§) indicates that the rhodanese sulfurtransferases Rhd‐TusA‐DsrE2 are also essential in the reaction mediated by this complex (Dahl, 2017). Apr, APS reductase; Asr, anaerobic sulfite reductase; Dox, thiosulfate:quinone oxidoreductase; Dsr, dissimilatory sulfite reductase; Fcc, flavocytochrome *c* sulfide dehydrogenase; Fsr, F_420_‐dependent sulfite reductase; Hdr, heterodisulfide reductase; Otr, octaheme tetrathionate reductase; Phs, thiosulfate reductase; Psr, polysulfide reductase; Qmo, quinone‐interacting membrane‐bound oxidoreductase; Sat, sulfate adenylyltransferase; Sir, sulfite reductase; Soe, sulfite‐oxidizing enzyme; SOR, sulfur oxygenase/reductase; Sor, sulfite‐acceptor oxidoreductase; Sox, sulfur‐oxidizing multienzyme complex; Sqr, sulfide:quinone oxidoreductase; Sre, sulfur reductase; SULT, sulfotransferase; Tet, tetrathionate hydrolase; Tsd, thiosulfate dehydrogenase; Ttr, tetrathionate reductase. [Color figure can be viewed at wileyonlinelibrary.com]

The biogeochemical sulfur cycle in DMW consists of abiotic and biologically mediated reactions (Fig. 2; Ehrlich *et al*., 2015), with the latter providing energy to many different microorganisms. Sulfate‐reducing bacteria (SRB) reduce sulfate (${\mathrm{SO}}_{4}^{2-}$) to sulfide (HS^−^), coupled to the oxidation of small organic compounds or H_2_ (Muyzer and Stams, 2008). Most of this sulfide is re‐oxidized by oxidized metals or sulfur‐oxidizing bacteria (SOB), either completely to sulfate (Jørgensen *et al*., 1991) or to different sulfur cycle intermediates (SCIs) including elemental sulfur (S^0^), polysulfides (${\mathrm{HS}}_{n}^{-}$), thiosulfate ($S_{2}O_{3}^{2-}$), tetrathionate ($S_{4}O_{6}^{2-}$) and sulfite (${\mathrm{SO}}_{3}^{2-}$; Zopfi *et al*., 2001; Kamyshny *et al*., 2011; Findlay, 2016). These SCIs can be used as electron donor or acceptor by various microorganisms including SRB and SOB (Rabus *et al*., 2013; Han and Perner, 2015; Dahl, 2017).

The detection and analysis of sulfur‐cycling genes, transcripts and proteins in DMW yield a powerful perspective on the diversity and activity of sulfur‐cycling microorganisms (Fig. 2), and more so when applied to metagenome‐assembled genomes (MAGs) or single‐cell amplified genomes (SAGs). Various ‘omics’ studies have yielded insight into the dominant SOB in DMW (Lavik *et al*., 2009; Walsh *et al*., 2009; Callbeck *et al*., 2018; Plominsky *et al*., 2018). However, only few studies have addressed the broader diversity of sulfur‐cycling microorganisms (Canfield *et al*., 2010; Stewart *et al*., 2012; Schunck *et al*., 2013; Hawley *et al*., 2014), without tapping into the larger potential of genome‐centric metagenomics and the available metagenome data. To fill this knowledge gap, we screened MAGs from DMW environments for sulfur‐cycling marker genes (Supporting Information Methods). Part of the MAGs was assembled from metagenomes of the Arabian Sea and ETSP OMZ cores produced by Tara Oceans (Parks *et al*., 2017; Tully *et al*., 2018). Other MAGs were assembled from metagenomes of 15 different water depths of the Black Sea (Villanueva *et al*., 2020; Suominen *et al*., 2019; Supporting Information Methods), which served as model for enclosed DMW environments.

Despite the central role of the sulfur cycle in DMW, current biogeochemical and microbiological knowledge has not been comprehensively reviewed so far. Therefore, we herein provide an overview of sulfur cycle processes in DMW, and we discuss the diversity, metabolism and physiology of the bacteria involved in these processes. In the following section, we will discuss current knowledge on SRB, who form an essential part of the sulfur cycle through the production of sulfide. The subsequent section treats SOB, covering well‐studied groups such as SUP05 and *Sulfurimonas*, less explored groups such as BS‐GSO2, and putative sulfur oxidizers such as SAR324 members. The final section discusses which bacteria could be involved in sulfur reduction or disproportionation.

## Sulfate‐reducing bacteria

The presence and activity of SRB in the dysoxic water column have been demonstrated through sulfate reduction rate measurements with isotopically labelled sulfate (^35^ ${\mathrm{SO}}_{4}^{2-}$) performed in euxinic settings such as the Black Sea with sulfate reduction rates up to 36 nmol l^−1^ day^−1^ (Sorokin, 1972; Jørgensen *et al*., 1991; Albert *et al*., 1995; Pimenov *et al*., 2000) and Mariager Fjord with rates up to 140 nmol l^−1^ day^−1^ (Sørensen and Canfield, 2004), but also in the ETSP OMZ core with rates up to 16.9 nmol l^−1^ day^−1^  (Canfield *et al*., 2010). More extensively conducted taxonomic marker studies point to a universal presence of SRB in DMW since the 16S rRNA genes of canonical SRB lineages of the class *Deltaproteobacteria* have been widely detected (Madrid *et al*., 2001; Vetriani *et al*., 2003; Lin *et al*., 2006; Fuchsman *et al*., 2011; Wright *et al*., 2012; Schunck *et al*., 2013; Ganesh *et al*., 2014; Rodriguez‐Mora *et al*., 2015; Suter *et al*., 2018; Callbeck *et al*., 2019).

### Presence and diversity

All known SRB reduce sulfate through the dissimilatory (bi)sulfite reductase (Dsr) pathway (Rabus *et al*., 2015). This consistency has facilitated functional marker investigations of the ecology of SRB. Such a functional marker approach is more appropriate than 16S rRNA gene surveys, as it does not require metabolic assumptions based on taxonomy. Although the core proteins of the Dsr pathway, Sat, AprBA and DsrAB, are also present in the reversed Dsr (rDsr) sulfur oxidation pathway (Dahl, 2017; Fig. 2), their reductive and oxidative versions are phylogenetically distinguishable (Meyer and Kuever, 2007; Loy *et al*., 2009; Müller *et al*., 2015; Pelikan *et al*., 2016). Reductive Dsr genes have been identified throughout suboxic and euxinic waters of the Black Sea (Neretin *et al*., 2007) and the Cariaco Basin (Rodriguez‐Mora *et al*., 2016), in sulfidic coastal waters off Peru (Schunck *et al*., 2013), in the core of the ETNP and ETSP OMZs (Canfield *et al*., 2010; Carolan *et al*., 2015), and in the Gdansk Deep within the Baltic Sea (Korneeva *et al*., 2015). Moreover, reductive Dsr genes were shown to be transcribed into mRNA (Stewart *et al*., 2012; Ulloa *et al*., 2012; Schunck *et al*., 2013; Rodriguez‐Mora *et al*., 2016; Saunders *et al*., 2019), providing evidence for activity of SRB. Although powerful, the results of Dsr markers should be cautiously interpreted (Anantharaman *et al*., 2018), as the Dsr pathway does not only facilitate dissimilatory sulfate reduction but can also mediate dissimilatory reduction and disproportionation of SCIs (Rabus *et al*., 2015; Florentino *et al*., 2017), and in some rare cases sulfur oxidation (Sigalevich and Cohen, 2000; Slobodkina *et al*., 2017; Thorup *et al*., 2017). Thus, although bacteria with reductive Dsr pathways are core members of the microbial community of DMW, their sulfur metabolism is not necessarily restricted to dissimilatory sulfate reduction. We therefore refer to them as putative SRB.

Surveys based on 16S rRNA or functional marker genes and metagenomic studies of DMW have revealed a high diversity of putative SRB, most of which are only distantly related to described species. The deltaproteobacterial putative SRB detected in 16S rRNA gene data sets are rarely affiliated with established genera (Fuchsman *et al*., 2011; Wright *et al*., 2012; Ganesh *et al*., 2014; Rodriguez‐Mora *et al*., 2015; Suter *et al*., 2018). Of all canonical SRB lineages, *Desulfobacteraceae* species are thought to be dominant due to the prevalence of their sequences in 16S rRNA data sets (Fuchsman *et al*., 2011; Wright *et al*., 2012; Rodriguez‐Mora *et al*., 2015; Suter *et al*., 2018) and metagenomic data sets (Canfield *et al*., 2010; Schunck *et al*., 2013). However, *Desulfobulbaceae* species have also been detected, specifically including the 16S rRNA genes of the genera *Desulfocapsa* and *Desulforhopalus* (Neretin *et al*., 2007; Canfield *et al*., 2010; Fuchsman *et al*., 2011; Fuchsman *et al*., 2012; Rodriguez‐Mora *et al*., 2015; Suter *et al*., 2018). Furthermore, bacteria related to the genus *Desulfatiglans* seem widespread in DMW since *Desulfatiglans*‐related sequences were retrieved from the Black Sea (16S rRNA genes; Vetriani *et al*., 2003; Neretin *et al*., 2007), coastal DMW off Peru (metagenomics; Schunck *et al*., 2013), the Gdansk Deep in the Baltic Sea (*dsrB* fragments; Korneeva *et al*., 2015) and the Cariaco Basin (*dsrA* fragments; Rodriguez‐Mora *et al*., 2016). Functional marker gene surveys indicated an even larger diversity of putative SRB beyond the *Deltaproteobacteria*, including *Thermodesulfovibrio*‐related bacteria in the ETSP OMZ core (Canfield *et al*., 2010) and diverse unknown putative SRB in euxinic basins (Korneeva *et al*., 2015; Rodriguez‐Mora *et al*., 2016).

**Fig 3:**
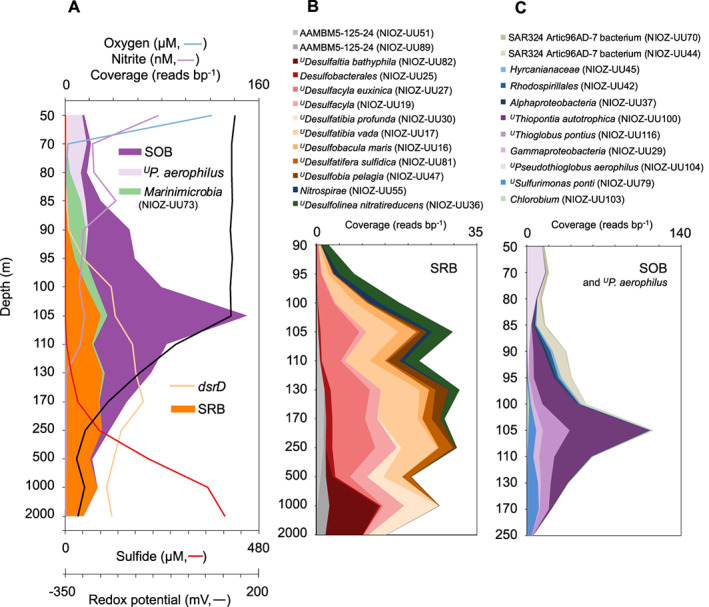
Black Sea water column distribution of metagenome‐assembled genomes (MAGs) of sulfur‐cycling bacteria based on their genetic capacity. A. Physicochemical measurements and normalized cumulative metagenome coverage of all MAGs of putative sulfur‐oxidizing bacteria (SOB) combined, ^*U*^ *P. aerophilus* (NIOZ‐UU104), *Marinimicrobia* (NIOZ‐UU73), all *dsrD* genes combined and all MAGs of putative sulfate‐reducing bacteria (SRB) combined in samples of 15 different depths of the Black Sea. The oxygen, nitrite and sulfide data correspond to the PHOXY cruise of June–July 2013 (Sollai *et al*., 2019). The Black Sea metagenome was also constructed from samples taken during this cruise as detailed in Supporting Information Methods and Villanueva and colleagues (2020). Redox potential was measured during the 64PE408 NESSC/SIAM cruise of January–February 2016 from samples with a closely agreeing sulfide profile (Fig. S1). B,C. Relative abundances of MAGs of (B) putative SRB and (C) putative SOB and ^*U*^ *P. aerophilus* were based on normalized metagenome coverage. See Supporting Information Methods for details on the methodology and data processing. [Color figure can be viewed at wileyonlinelibrary.com]

**Fig 4:**
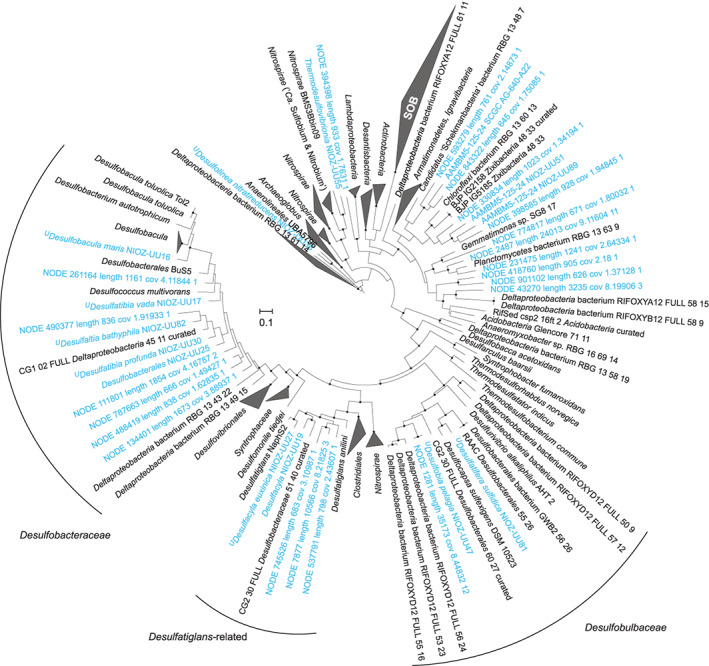
Maximum‐likelihood phylogenetic reconstruction based on bacterial reductive DsrA proteins predicted from Black Sea MAGs and unbinned contigs (blue) and reference genomes (Anantharaman *et al*., 2018). Black dots indicate support of > 95% out of 1,000 ultra‐fast bootstraps. The scale bar indicates substitutions per site. See Supporting Information Methods for methodology and Data S1 for the full phylogenetic tree in Newick format. [Color figure can be viewed at wileyonlinelibrary.com]

The *dsrD* gene, encoding a small protein with a possible regulatory function (Mizuno *et al*., 2003; Venceslau *et al*., 2014), is an alternative functional marker gene for detection of SRB (Mussmann *et al*., 2005). It has been used to investigate bacterial genomes with *dsrAB* genes lacking a clear oxidative/reductive affiliation (Anantharaman *et al*., 2018), since *dsrD* forms a reliable marker for the reductive Dsr pathway when present together with other Dsr genes (Rabus *et al*., 2015). We detected the *dsrD* gene – in the context of other *dsr* genes – in metagenomes and MAGs from the Black Sea throughout the euxinic and suboxic zones (Fig. 3A,B), confirming previously reported distributions of putative SRB (Neretin *et al*., 2007). We could not obtain any assembled reductive *dsrA* genes from publicly available OMZ metagenomes (Canfield *et al*., 2010; Ganesh *et al*., 2014; Fuchsman *et al*., 2017; Tully *et al*., 2018; Saunders *et al*., 2019), likely due to sampling bias (see following subsection) and insufficient sequencing depth. However, many complete *dsrA* genes could be retrieved from the Black Sea metagenome (Supporting Information Methods). An analysis of these *dsrA* sequences supported the view emerging from previous studies: a large diversity of putative SRB, with a somewhat distant relationship to canonical SRB belonging to *Desulfobacula*, *Desulfococcus*, *Desulfocapsa*, *Desulfatiglans* and *Thermodesulfovibrio*, and to non‐canonical lineages other than the *Deltaproteobacteria* or *Nitrospirae* (Fig. 4). This view mirrors the overly large diversity of SRB that can generally be found in marine sediments (Muyzer and Stams, 2008; Müller *et al*., 2015). Despite the wealth of knowledge on the metabolism of SRB, the ecophysiological causes behind this diversity are currently poorly understood.

### Physiology and metagenomics

We have a considerable understanding of the physiology of SRB from marine sediments, owing to a rich diversity of isolated SRB that are available for laboratory research (Muyzer and Stams, 2008; Rabus *et al*., 2015). In contrast, no SRB have been isolated from DMW, except for two subspecies of *Desulfovibrio oceani* from the ETSP OMZ (Finster and Kjeldsen, 2010). It can be reasonably assumed that most of the deltaproteobacterial putative SRB detected in DMW adhere to the general metabolism of dissimilatory reduction of sulfate and oxidation of small organic compounds or H_2_. However, the lack of closely related described SRB does not allow further constraining of metabolic niches based on taxonomic affiliation, nor does it allow hypotheses on the many other variable physiological aspects.

**Fig 5:**
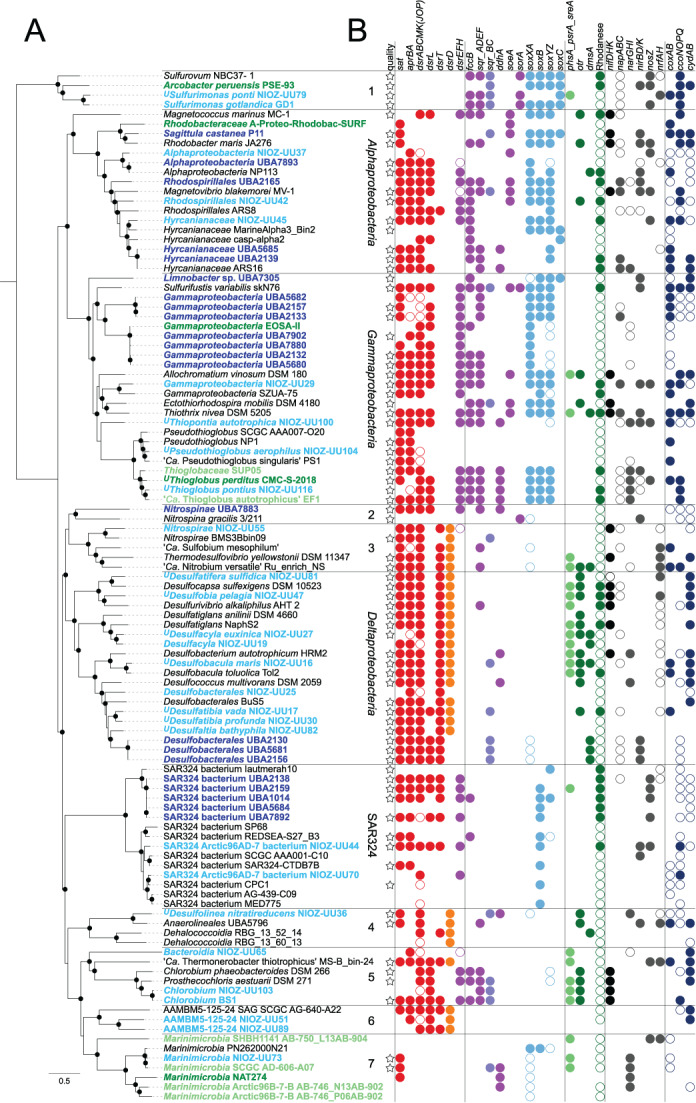
A phylogenomic and genetic overview of important microbial players in the marine sulfur cycle of dysoxic marine water (DMW) environments. A. An unrooted phylogenomic maximum‐likelihood tree constructed from a concatenated alignment of 120 single‐copy household genes (Supporting Information Methods). Phylogenetic clades were identified, with numbers indicating the following lineages: 1, *Campylobacterota*; 2, *Nitrospinae*; 3, *Nitrospirae*; 4, *Chloroflexi*; 5, *Bacteroidetes*; 6, candidate phylum AAMBM5‐125‐24; 7, *Marinimicrobia*. Black dots indicate support by > 95% out of 1,000 ultra‐fast bootstraps. The scale bar indicates substitutions per site. The tree includes data from all available genomes (March 2020) from DMW sites (bold, coloured by environment following the colour code of Fig. 1) that contain dissimilatory sulfur genes, and relevant reference genomes (black). The superscript prefix ‘*U*’ indicates uncultured species for which a taxonomy has been proposed based on a high‐quality genome, functional annotation and environmental distribution (Konstantinidis *et al*., 2017) with the genome sequences as type material (Chuvochina *et al*., 2019; Murray *et al*., 2020; Supporting Information Protologue). B. An overview of the presence of functional genes enabling conversions of sulfur, nitrogen and oxygen, following the colour scheme of Fig. 2. Shortly, red indicates core genes of the Dsr/rDsr pathways, orange indicates *dsrD*, purple indicates *dsrEFH* and various oxidative sulfur genes, light blue indicates *sox* genes, light‐green indicates *phs*/*psr*/*sre* genes, dark‐green indicates various (potentially) reductive sulfur genes, black/dark grey indicates nitrogen genes, dark blue indicates oxygen reduction genes. The presence of the indicated functional genes or gene clusters is shown with filled circles; open circles reveal incomplete gene clusters. For ‘Rhodanese’, filled circles indicate 10 or more rhodanese domains (Supporting Information Methods). Stars distinguish the high‐quality genomes (> 80% complete, < 5% contaminated) from the medium‐quality genomes (> 50% complete, < 10% contaminated) analysed. Only two low‐quality metagenome‐assembled genomes, that is, that of *Dehalococcoidia* RBG_13_52_14 (35% complete, 2% contaminated) and the population genome of *Gammaproteobacteria* EOSA‐II composed of multiple combined single‐cell amplified genomes (63% complete, 21% contaminated), were also included. A comprehensive overview of genome origin, quality, classification, annotation and average amino acid identity (AAI) between genomes can be found in Table S2. [Color figure can be viewed at wileyonlinelibrary.com]

These challenges can be addressed by genome‐centric metagenomics, exemplified by recent explorations of the potential metabolism of *Desulfatiglans*‐related SAGs from marine sediments (Jochum *et al*., 2018) and of the identity and potential metabolism of non‐canonical putative SRB from various environments (Wasmund *et al*., 2016; Anantharaman *et al*., 2018; Hausmann *et al*., 2018; Thiel *et al*., 2018; Meier *et al*., 2019). With these aims, we mined metagenome data to obtain 13 MAGs of putative SRB from the Black Sea, encoding complete or incomplete reductive Dsr pathways (Figs 3A,B and 5, Table S2, Supporting Information Methods). In agreement with previous diversity studies, most of the putative SRB MAGs were affiliated with *Desulfobacterales*, *Desulfobulbales* and *Nitrospirae* but did not classify within established genera, except for ^*U*^ *Desulfobacula maris*. Four of the putative SRB MAGs from the Black Sea (52%–93% complete, 1%–3% contaminated) affiliated with the non‐canonical phyla *Nitrospirae*, *Chloroflexi* and candidate phylum AAMBM5‐125‐24. Other novel putative SRB within the phylum *Nitrospirae*, ‘*Candidatus* Sulfobium mesophilum’ (Zecchin *et al*., 2018) and ‘*Candidatus* Nitrobium versatile’ (Arshad *et al*., 2017) were only distantly related to *Nitrospirae* MAG NIOZ‐UU55 [< 51% amino acid identity (AAI), Fig. 5, Table S2]. Another phylogenetically related SAG containing sulfate‐reducing genes (AAMBM5‐125‐24) has been retrieved from the euxinic Zodletone Spring (Fig. 5; Youssef *et al*., 2019). Despite the novelty revealed by this genome‐centric approach, it should be noted that it did not encompass the complete diversity of putative SRB in the Black Sea detected according to the *dsrD* (Fig. 3A) and *dsrA* diversity (Fig. 4).

More so than taxonomic affiliation, functional gene annotation offers insight into the possible energy metabolism(s) of these microorganisms. Metagenome mining yielded three *Desulfobacterales* MAGs from the Arabian Sea OMZ core (95%–96% complete, 0.7% contaminated) with a complete Dsr pathway but lacking the reductive marker *dsrD* (Fig. 5, Table S2, Supporting Information Methods). Moreover, they harbored oxidative instead of reductive *dsrA* genes possibly horizontally transferred from *Chlorobia* or SAR324 bacteria (Data S1). Together with the absence of *dsrD* and the presence of *sqr* (Fig. 5), this suggests a sulfur‐oxidizing rather than sulfate‐reducing metabolism. These MAGs thus question the common assumption that all *Desulfobacterales* reduce sulfate, and undermine taxonomy‐based physiological assumptions in general. Habitat profiling can further support metabolic hypotheses based on functional annotation of the genes in different MAGs. For instance, the SRB that reside exclusively in the deeper euxinic waters of the Black Sea (^*U*^ *Desulfatibia profunda*, ^*U*^ *Desulfaltia bathyphila*, *Desulfacyla* NIOZ‐UU19; 95%–97% complete) apparently lack the genes to detoxify oxygen (*cydAB*) or hydrogen peroxide (catalase; Table S2) and to utilize alternative electron acceptors (Figs 3B and 5), reflecting their probably purely euxinic and strictly sulfate‐reducing lifestyle. In contrast, the MAGs of SRB relatively abundant in suboxic waters (^*U*^ *Desulfolinea nitratireducens*, *Nitrospirae* NIOZ‐UU55, ^*U*^ *Db. maris*, ^*U*^ *Desulfatibia vada*, ^*U*^ *Desulfacyla euxinica*; 73%–93% complete, 1%–4% contaminated) encode a plethora of genes for the energy‐conserving reduction of alternative electron acceptors such as S^0^ or thiosulfate (*psrA*/*phsA*/*sreA*), tetrathionate (*otr*), nitrate (*napABC*, *narGHI*) and nitrite (*otr*, *nirBD*, *nirK*, *nrfAH*). They also encode terminal oxidases (*coxAB*, *ccoNOPQ*, *cydAB*; Figs 3B and 5), which could be part of complete oxygen respiratory chains. These metabolic potentials are in line with a complex sulfur cycle interlinked with nitrogen cycling and oxygen intrusions (see the following sections). Whether the same SRB species as herein detected in the Black Sea are also present in other DMW requires additional research. However, the metagenomic data from the Black Sea offer a basis for such investigations ranging from 16S rRNA surveys to genome‐centric genomics. Expression studies are required to investigate the metabolism of SRB in DMW, and whether they shift their metabolism in response to changing conditions. For instance, the expression of *Desulfocapsa*‐related nitrogen fixation (*nif*) genes in the suboxic and upper euxinic zones of the Black Sea (Kirkpatrick *et al*., 2018) suggests that the *nif*‐encoding SRB ^*U*^ *Desulfatifera sulfidica* (99% complete, 2% contaminated) and ^*U*^ *Desulfobia pelagia* (96% complete, no contamination) could be actively fixing nitrogen. These genes are also encoded by *Nitrospirae* NIOZ‐UU55 (73% complete, 1% contaminated). This supports a growing body of evidence for nitrogen fixation by putative SRB in DMW (Jayakumar *et al*., 2012; Bonnet *et al*., 2013; Loescher *et al*., 2014; Christiansen and Loescher, 2019).

### Particles as microhabitat

Sulfate is thermodynamically an inferior electron acceptor to nitrate and nitrite (Table S3), implying that denitrifying microorganisms will outcompete SRB for electron donors in suboxic waters and OMZ cores. How then is dissimilatory sulfate reduction sustained, especially in nitrite‐ and nitrate‐rich OMZ cores? It has been postulated that SRB occupy microhabitats inside organic particles, in which nitrate and nitrite have already been depleted (Fuchsman *et al*., 2011; Wright *et al*., 2012). These organic particles or ‘marine snow’ (Alldredge and Silver, 1988) are particularly abundant in suboxic waters (Karl and Knauer, 1991; Taylor *et al*., 2001; Sorokin, 2002) and OMZs (Whitmire *et al*., 2009; Roullier *et al*., 2014), compared to other regions of the oceanic water column. When oxygen levels drop below ~25 μM, particles of the predominant size range (100–200 μM in diameter; Roullier *et al*., 2014) develop inner anoxic microhabitats due to limitation of oxygen diffusion (Shanks and Reeder, 1993; Klawonn *et al*., 2015; Ploug and Bergkvist, 2015). This implies that in nitrate‐rich dysoxic waters, particles will develop a nitrate‐depleted core, as nitrate rarely exceeds a concentration of 25 μM in DMW and diffuses slower than oxygen (Fuchsman *et al*., 2019). Thus, sulfate‐reducing microhabitats may be abundant in non‐sulfidic DMW.

SRB in seawater seem to be more abundant in particles than in free suspension. The 16S rRNA gene sequences of canonical deltaproteobacterial SRB were found to be predominantly particle‐associated (> 30 μm) in marine suboxic zones (Fuchsman *et al*., 2011; Suter *et al*., 2017, 2018) and the ETNP OMZ core (Fuchsman *et al*., 2017). Moreover, reductive *dsrA* genes in the ETNP OMZ core were almost exclusively detected in the particle (> 30 μm) fraction, whereas oxidative *dsrA* genes showed no specific particle association (Saunders *et al*., 2019). This particle‐bound lifestyle causes SRB to be significantly underrepresented in some molecular ecological studies. It is common practice to employ a pre‐filter step for the collection of biomass to remove eukaryotes (1.6–10 μM pore size cut‐off), thus also removing particles and particle‐associated SRB. This methodology has been applied for investigations in DMW (Canfield *et al*., 2010; Stewart *et al*., 2012; Ulloa *et al*., 2012; Ganesh *et al*., 2014; Hawley *et al*., 2014), which may explain why SRB sequences were present in low abundance or absent. In contrast, studies omitting a pre‐filter have identified SRB sequences in substantial proportion (Neretin *et al*., 2007; Fuchsman *et al*., 2011; Carolan *et al*., 2015; Rodriguez‐Mora *et al*., 2016; Saunders *et al*., 2019). Particle sinking in standard Niskin sampling bottles also caused bias against particles and SRB sequences (Suter *et al*., 2017). Thus, complete circumvention of these potential biases requires *in situ* filtration devices, which have occasionally been used in DMW (Lavik *et al*., 2009; Marschall *et al*., 2010; Sollai *et al*., 2019). *In situ* filtration was also applied for obtaining the Black Sea MAGs presented herein, indeed resulting in higher estimated relative abundances of SRB than found by Neretin and colleagues (2007) in both the suboxic zone (< 13% vs. < 2% of all bacteria) and the euxinic zone (< 20% vs. < 5%, Supporting Information Methods). Since these estimated fractional abundances may still be biased by DNA extraction methods, ideally an extraction‐independent method should also be applied such as fluorescence *in situ* hybridization of functional genes (Barrero‐Canosa *et al*., 2017). To achieve comparability between different studies, similar sampling methodology with minimal bias is essential and should be carefully evaluated.

## Sulfur‐oxidizing bacteria

The oxidative part of the sulfur cycle starts with the competition between SOB and abiotic reactions for sulfide (Luther *et al*., 2011). Depending on the sulfide oxidation route, a range of possible sulfide oxidation products can be formed (Fig. 2), having a significant effect on the rest of the biogeochemistry in DMW. Oxidized metals such as manganese oxide (MnO_2_) or ferric (oxy)hydroxides are so efficient in catalyzing sulfide oxidation (Yao and Millero, 1993; Ma *et al*., 2006) that even at micromolar concentrations MnO_2_ is thought to be abiotically responsible for the bulk of the sulfide oxidation in systems with broad stable chemoclines and low oxygen flux such as the Black Sea (Jørgensen *et al*., 1991; Konovalov *et al*., 2003; Trouwborst *et al*., 2006; Stanev *et al*., 2018) and Cariaco Basin (Ho *et al*., 2004). Chemical oxidation of sulfide produces SCIs such as S^0^ and thiosulfate, which are commonly detected in euxinic marine waters (Jørgensen and Bak, 1991; Zopfi *et al*., 2001; Li *et al*., 2008; Kamyshny *et al*., 2013; Findlay *et al*., 2014). Like sulfide, SCIs can be converted by SOB to sulfate as energy source.

Microorganisms that oxidize sulfur compounds possess widely differing metabolisms, including autotrophy or heterotrophy, and chemotrophy or phototrophy (Dahl, 2017). Some SOB oxidize a wide range of sulfur compounds as primary energy source, whereas others facultatively oxidize specific sulfur compounds such as thiosulfate as supplementary energy source (Sorokin, 2003). Photolithotrophic SOB – green or purple sulfur bacteria – can become dominant if euxinic marine waters overlap with the photic zone in shallow waters (Findlay *et al*., 2015; Pjevac *et al*., 2015; Findlay *et al*., 2017; Pjevac *et al*., 2019). The deeper the chemocline, the smaller the population and role of phototrophic SOB, exemplified by low‐light‐adapted *Chlorobium* bacteria in the Black Sea (approximately 100 m depth; Overmann *et al*., 1992; Manske *et al*., 2005; Marschall *et al*., 2010). They are outnumbered by chemolithotrophic SOB, with gammaproteobacterial SUP05 bacteria (Lavik *et al*., 2009; Canfield *et al*., 2010; Glaubitz *et al*., 2013) and sulfur‐oxidizing *Campylobacterales* bacteria such as *Sulfurimonas* species (Grote *et al*., 2008; Schunck *et al*., 2013; Callbeck *et al*., 2019) as foremost examples. However, the biogeochemical impact on element cycling is not always related to cellular abundance (Pester *et al*., 2012; Hausmann *et al*., 2019), exemplified by magnetotactic *Magnetococcus*‐related bacteria that shuttle the scarcely available phosphate from the Black Sea chemocline into the euxinic zone (Schulz‐Vogt *et al*., 2019), which suggests a sulfur‐oxidizing physiology akin to other *Magnetococcus* species (Bazylinski *et al*., 2013). This underscores the importance of using multiple approaches when studying functional groups of microorganisms, including sulfur‐cycling bacteria. Here, we will mainly discuss well‐studied chemolithotrophic SOB specifically abundant in OMZ core waters and other deep DMW.

### SUP05 bacteria

Based on 16S rRNA gene surveys, specific gammaproteobacterial bacteria belonging to the SUP05 clade and closely related to known sulfur‐oxidizing symbionts have been identified as abundant putative SOB in DMW (reviewed by Wright *et al*., 2012). The capacity of these bacteria for chemolithoautotrophic nitrate reduction – most probably coupled to the oxidation of sulfide and/or SCIs – in DMW was strongly indicated by stable isotope probing experiments with labelled inorganic carbon (Grote *et al*., 2008; Glaubitz *et al*., 2010) and correlations with rate measurements of nitrate reduction and dark carbon fixation (Lavik *et al*., 2009; Schunck *et al*., 2013). Direct cell counts with fluorescent probes showed that SUP05 bacteria may form a dominant group of the microbial community; they comprised up to 50% of the microbial population in euxinic shelf waters off Namibia and Peru (< 3 × 10^6^ cells ml^−1^; Lavik *et al*., 2009; Callbeck *et al*., 2018), up to 17% in the ETSP OMZ core (5 × 10^5^ cells ml^−1^; Callbeck *et al*., 2018), up to 30% at an oxic–euxinic interface in the Baltic Sea (4 × 10^5^ cells ml^−1^, Landsort Deep) and up to 10% and 13% in the suboxic and euxinic zone of the Black Sea respectively (< 7 × 10^4^ cells ml^−1^; Glaubitz *et al*., 2013).

The presumed physiology of SUP05 bacteria was supported by the presence in their MAGs of genes for sulfide oxidation (*sqr*, *fccAB*), the ‘Sox’ thiosulfate oxidation pathway (*soxXABYZ*), the rDsr sulfur oxidation pathway (*sat*, *aprBA*, *dsrABCMK*, *dsrEFH*; Figs 2 and 5), nitrate reduction (*narGHIJ*) and inorganic carbon fixation through the Calvin–Benson–Bassham cycle (Walsh *et al*., 2009; Canfield *et al*., 2010; Murillo *et al*., 2014; Callbeck *et al*., 2018). This physiology has been confirmed by cultivation experiments of the only current SUP05 isolate, '*Candidatus* Thioglobus autotrophicus’ (Shah *et al*., 2017), which showed growth with sulfide, thiosulfate, thiotaurine and stored S^0^ as energy source (Shah *et al*., 2019). The four available SUP05 genomes from DMW show variation in the presence of other nitrogen‐respiration genes (*nirK*, *nirS*, *nirBD*, *norCB*, *nosZ*; Fig. 5) and oxidative phosphorylation genes (*coxBAC*, *ccoNOPQ*, cytochrome *bc*_1_ complex genes; Fig. 5), indicating metabolic diversification of the strains within this clade. Corresponding with their high abundance, SUP05 bacteria generally dominate the detection of rDsr pathway genes, transcripts and proteins in DMW (Canfield *et al*., 2010; Stewart *et al*., 2012; Hawley *et al*., 2014). The sister clade ARCTIC96BD‐19 is represented by ‘*Candidatus* Thioglobus singularis’ (Marshall and Morris, 2013), but ARCTIC96BD‐19 genomes are too divergent from SUP05 genomes to consider them the same genus (63%–66% AAI, Table S2). ‘*Ca*. T. singularis’ has an organoheterotrophic aerobic lifestyle and does not oxidize sulfur (Spietz *et al*., 2019). These features are probably representative of all ARCTIC96BD‐19 bacteria, based on the absence of most sulfur oxidation genes in their genomes (Swan *et al*., 2011; Fig. 5) and a preference for oxic waters (Wright *et al*., 2012; Fig. 3A; *^U^P. aerophilus*). To reflect their distinct taxonomy and physiology, we suggest to rename the ARCTIC96BD‐19 clade to *Pseudothioglobus* (Supporting Information Protologue).

The affinity of SUP05 bacteria for sulfide is higher than reported for any other bacterium or substrate (Crowe *et al*., 2018), demanding a re‐evaluation of the existing definition of euxinia. Currently, the sulfide concentration threshold to distinguish non‐sulfidic from euxinic conditions commonly falls in the range of 0.5–1 μM. This threshold is similar to the in vitro Michaelis–Menten half‐saturation constant (*K*_*m*_) of 2 μM of purified high‐affinity Sqr proteins (Schutz *et al*., 1997; Brito *et al*., 2009) and the *K*_*m*_ found for phototrophic SOB (> 0.8 μM; Van Gemerden, 1984). However, the estimated *K*_*m*_ of SUP05 bacteria is much lower (25–340 nM; Crowe *et al*., 2018). The most widely used method for determining sulfide concentrations has a sulfide detection limit of 0.1 μM (Cline, 1969; Jørgensen *et al*., 1991; Zopfi *et al*., 2001), hence falling within this estimated range. Thus, we suggest it is biologically sound and practically feasible to use a sulfide threshold of at most 0.1 μM to define euxinia, at least in marine environments. However, an even lower threshold would be more accurate, as SUP05 bacteria consume sulfide at < 5 nM (Crowe *et al*., 2018). Such low concentrations can be detected and quantified with sensitive voltammetric sensors (Luther *et al*., 1991; Luther *et al*., 2008). The findings of Crowe and colleagues (2018) illustrate the value of studies that quantify properties such as substrate affinity and should motivate further investigation, for instance with respect to sulfide toxicity or substrate affinity for sulfide of other key SOB. Such studies are required to advance biogeochemical models that explicitly take microbial community composition and function into account by integrating omics data (Reed *et al*., 2014; Louca *et al*., 2016).

### Campylobacterota

The SOB of the phylum *Campylobacterota* (formerly *Epsilonproteobacteria*; Waite *et al*., 2017, 2018) are more diverse and environment‐specific than the common SUP05 bacteria. The most widespread *Campylobacterota* genus in DMW is *Sulfurimonas*. Members of this genus dominate the upper euxinic zone of the Black Sea and Baltic Sea at a count of 15% to 30% of all microorganisms (< 2 × 10^5^ cells ml^−1^) and outnumber SUP05 bacteria (Brettar *et al*., 2006; Grote *et al*., 2008; Glaubitz *et al*., 2010). Members of another *Campylobacterota* genus, *Arcobacter*, are generally less abundant in euxinic basins (Glaubitz *et al*., 2008; Fuchsman *et al*., 2012; Rodriguez‐Mora *et al*., 2013), but proliferate in euxinic shelf waters during sulfidic events (< 25% of all cells; < 1 × 10^6^ cells ml^−1^; Lavik *et al*., 2009; Schunck *et al*., 2013; Callbeck *et al*., 2019). Four *Campylobacterota* isolates have thus far been obtained from DMW, all facultative anaerobes capable of sulfur oxidation and nitrate reduction: *Sulfurimonas gotlandica* (Grote *et al*., 2012) and ‘*Candidatus* Sulfurimonas baltica’ (Henkel, 2019), both from a redoxcline in Gotland Basin; ‘*Candidatus* Sulfurimonas marisnigri’ from the Black Sea euxinic zone (Henkel *et al*., 2019); and *Arcobacter peruensis* from euxinic coastal waters off Peru (Callbeck *et al*., 2019). *A. peruensis* was only demonstrated to use sulfide as energy source (Callbeck *et al*., 2019), whereas the *Sulfurimonas* species were grown with sulfide, SCIs and H_2_ (Labrenz *et al*., 2013; Henkel, 2019). As is common in *Campylobacterota* members, the genomes of *S. gotlandica* and *A. peruensis* lack rDsr genes and these SOB are presumed to oxidize sulfur compounds through a variant of the Sox pathway encoded by two operons (*soxXY*_*1*_ *Z*_*1*_ *AB* and *soxCDY*_*2*_ *Z*_*2*_; Meier *et al*., 2017; Pjevac *et al*., 2018; Götz *et al*., 2019; Figs 2 and 5). We recovered a *Sulfurimonas* MAG from the Black Sea (^*U*^ *Sulfurimonas ponti*), which also lacks most Sox genes despite being virtually complete (97% completeness, 4% contamination, Fig. 5). ^*U*^ *S. ponti* may oxidize sulfide incompletely to S^0^, or use an alternative route such as the Hdr‐like sulfur oxidation pathway (Boughanemi *et al*., 2016).

The heterotrophic *A. peruensis* was not capable of fixing inorganic carbon and requires an organic carbon source such as acetate (Callbeck *et al*., 2019), whereas the autotrophic *Sulfurimonas* species fixed inorganic carbon for growth, presumably through the reverse tricarboxylic acid cycle (Grote *et al*., 2008; Henkel, 2019). In euxinic coastal waters, sufficient organic carbon can be available to allow *A. peruensis* to successfully compete for sulfur substrate with autotrophic SOB by achieving a higher carbon assimilation rate and therefore probably also a higher growth rate (Callbeck *et al*., 2019). *Sulfurovum* species of the *Campylobacterota* phylum were highly abundant during sulfidic events in coastal waters (Lavik *et al*., 2009; Schunck *et al*., 2013; Callbeck *et al*., 2019) and possibly outnumber *Sulfurimonas* in Cariaco Basin (Rodriguez‐Mora *et al*., 2013; Rodriguez‐Mora *et al*., 2016; Taylor *et al*., 2018). Previous cultivation‐ and metagenomics‐based studies of *Sulfurovum* members have primarily addressed hydrothermal vent habitats. They revealed metabolic similarity to *Sulfurimonas* species with respect to sulfur oxidation, carbon fixation, and nitrate reduction (Yamamoto *et al*., 2010; Giovannelli *et al*., 2016; Jeon *et al*., 2017; Meier *et al*., 2017; Mori *et al*., 2018). However, one notable exception is *Sulfurovum aggregans*, which cannot oxidize sulfur but instead reduces it (Mino *et al*., 2014). As such, multiple biochemical roles are possible for *Sulfurovum* species in DMW.

Sulfur‐oxidizing autotrophs such as *Sulfurimonas* and SUP05 bacteria compete for very similar niches through different strategies. The metabolically specialized, streamlined (< 1.5 Mbp genomes) and non‐motile SUP05 bacteria prefer stable conditions, while the motile and more adaptable *Sulfurimonas* species benefit from a less stable chemocline with more mixing of sulfide, nitrate and oxygen (Rogge *et al*., 2017; Taylor *et al*., 2018). Furthermore, SUP05 bacteria are most abundant at low‐sulfidic conditions (< 5 μM; Glaubitz *et al*., 2013; Rogge *et al*., 2017), which may be due to their unparalleled high affinity for sulfide (Crowe *et al*., 2018) and their capability to store S^0^ for later usage when external substrates are absent (Shah *et al*., 2019). In euxinic basins, *Sulfurimonas* species can thrive simultaneously with SUP05 bacteria, but have a relative abundance peak in slightly deeper, more sulfidic waters (median 17 μM; Fig. 3C; Rogge *et al*., 2017). Here, the electron acceptors oxygen and nitrate are irregularly available (Konovalov *et al*., 2003; Glaubitz *et al*., 2010; Glaubitz *et al*., 2013). *Sulfurimonas* species have adapted to these conditions through motility and chemotaxis towards nitrate‐rich conditions (Grote *et al*., 2012), which is sustained by energy storage in the form of polyphosphate (Möller *et al*., 2019). Furthermore, *Sulfurimonas* species probably conserve more energy from nitrate than SUP05 bacteria, since instead of partial denitrification to nitrite (Shah *et al*., 2017) or possibly nitrous oxide (Walsh *et al*., 2009; Hawley *et al*., 2014), *S. gotlandica* can perform complete denitrification to nitrogen gas (Labrenz *et al*., 2013) and ^*U*^ *S. ponti* could perform ammonification (*nrfAH*, Fig. 5).

Intriguingly, ‘*Ca*. S. marisnigri’ is the first bacterium demonstrated to couple sulfur oxidation to the reduction of MnO_2_ to Mn^2+^ for growth (Henkel *et al*., 2019). This trait could be highly beneficial in euxinic basins such as the Black Sea since in contrast to oxygen and nitrate, MnO_2_ is in constant supply – albeit at low concentrations – since it is particulate and sinks (Tebo, 1991; Konovalov *et al*., 2004; Trouwborst *et al*., 2006). This metabolic capacity could also answer the long‐pending question of how high carbon fixation rates are sustained in euxinic waters without sufficient nitrate, nitrite or oxygen (Jørgensen *et al*., 1991; Taylor *et al*., 2001; Ho *et al*., 2004; Jost *et al*., 2010; Kirkpatrick *et al*., 2018). Indeed, a reaction–diffusion model by Yakushev and colleagues (2007) required coupling of MnO_2_ reduction to carbon fixation to reproduce the observed chemical profiles. There are indications that the reaction rates of abiotic and microbial sulfide oxidation by Mn in euxinic basins are in the same order of magnitude (Jørgensen *et al*., 1991; Sorokin *et al*., 1995; Henkel, 2019). However, there is currently no insight into the *in situ* abundance of ‘*Ca*. S. marisnigri’. Like *S. gotlandica* (Grote *et al*., 2012), it does not affiliate with the locally abundant *Sulfurimonas* GD17 subclade (95%–96% 16S rRNA gene sequence similarity). Further investigation through molecular studies is currently challenging, as the enzymatic pathway allowing MnO_2_ reduction is unknown. Nevertheless, these findings have large consequences for our view on euxinic biogeochemistry, as sulfide‐driven denitrification and nitrogen loss may effectively be bypassed. Mn‐dependent sulfide oxidation could even result in a fixed nitrogen gain, since both *S. marisnigri* and *S. baltica* can apparently fix nitrogen (Henkel, 2019), confirming a recently published hypothesis (Kirkpatrick *et al*., 2018).

### Other sulfur‐oxidizing lineages

As described above, the physiology of some of the key SOB has been explored in some detail, but other microbial players are waiting to be described. Predominantly genomic studies point to a wide phylogenetic diversity of poorly studied SOB for which important ecological or biogeochemical roles in DMW have been demonstrated or are strongly indicated. The class *Gammaproteobacteria* probably contains relevant SOB that do not affiliate with the SUP05 clade, notwithstanding their key role. Firstly, genomes of the EOSA‐II lineage were retrieved from coastal waters and the OMZ in Southern Pacific waters (Fig. 5), actively expressing sulfur oxidation genes in the ETSP OMZ core (Plominsky *et al*., 2018). Secondly, the gammaproteobacterial BS‐GSO2 clade was detected in the Black Sea as autotrophic lineage with a peak in relative abundance at the euxinic interface together with SUP05, suggesting it uses sulfur as energy source (Glaubitz *et al*., 2010). This clade is especially noteworthy since sequencing studies indicate that BS‐GSO2 bacteria may outnumber SUP05 bacteria in the Black Sea (Fuchsman *et al*., 2011; Kirkpatrick *et al*., 2018; Fig. 3C) and Cariaco Basin (Suter *et al*., 2018; Taylor *et al*., 2018). The only currently available BS‐GSO2 MAG is that of ^*U*^ *Thiopontia autotrophica* obtained from the Black Sea metagenomes analyzed herein (NIOZ‐UU100, 93% complete, 0.1% contamination) with 99% 16S rRNA gene identity with the original BS‐GSO2 sequence reported by Glaubitz and colleagues (2010). Indeed, ^*U*^ *T. autotrophica* possesses the genes for sulfur oxidation (rDsr) and the Calvin–Benson–Bassham cycle, but it differs from SUP05 bacteria in lacking most Sox genes and encoding a complete denitrification pathway (Fig. 5). It thus seems the BS‐GSO2 clade has been overshadowed by SUP05, yet may successfully compete for the same niche.

Bacteria of the uncultivated SAR324 candidate phylum have been abundantly and ubiquitously detected in DMW (Fuchsman *et al*., 2011; Wright *et al*., 2012; Beman and Carolan, 2013; Lüke *et al*., 2016; Suter *et al*., 2018; Fig. 3C). These SAR324 bacteria encode the rDsr sulfur oxidation pathway, which could enable them to oxidize sulfur for energy (Swan *et al*., 2011; Sheik *et al*., 2014; Fig. 5). Notably, they may couple this process to the reduction of the greenhouse gas nitrous oxide as they encode nitrous oxide reductase genes (*nosZ*, Fig. 5). The uncultured alphaproteobacterial family *Hyrcanianaceae* also harbors putative SOB with genomes retrieved from hydrothermal vent plumes (Zhou *et al*., 2020), the Arabian Sea OMZ core, and the Black Sea (Fig. 5). Low‐abundance SOB could still significantly alter their environment, for instance through diazotrophy or N_2_ fixation. Examples of such SOB are the heterotrophic alphaproteobacterium *Sagitulla castanea* isolated from euxinic Peru shelf waters (Martínez‐Pérez *et al*., 2018) or photolithoautotrophic *Chlorobium* strains (Overmann *et al*., 1992; Manske *et al*., 2005; Marschall *et al*., 2010; *nifDHK*; Figs 3C, 5). Finally, marine *Nitrospinae* members have been shown to oxidize nitrite (Lücker *et al*., 2013; Sun *et al*., 2019; Kitzinger *et al*., 2020), but the presence of a complete rDsr pathway in a *Nitrospinae* MAG from the ETSP OMZ core (UBA7883, 97% complete, 1% contaminated) opens up the possibility that some members may use sulfur as additional or alternative energy source (Fig. 5).

## Sulfur‐reducing and sulfur‐disproportionating bacteria

The SCIs formed or introduced in DMW could form the substrate for further oxidation by SOB, but could also be used as electron acceptor by sulfur‐reducing microorganisms, or as substrate for disproportionation (Fig. 2), thus shortcutting the sulfur cycle as has been suggested for other aquatic ecosystems (Tonolla *et al*., 2004; Wilbanks *et al*., 2014; Bhatnagar *et al*., 2020). Similar to abiotic sulfide oxidation, SOB may introduce SCIs in DMW through oxidation of sulfide to S^0^ (Dahl, 2017), which is stored intracellularly by the abundant SUP05 bacteria (Shah *et al*., 2019). Part of this S^0^ may be released into the environment due to grazing of SUP05 bacteria by protists (Lin *et al*., 2007; Glaubitz *et al*., 2008; Anderson *et al*., 2013) or due to lysis by SUP05‐infecting viruses (Cassman *et al*., 2012; Anantharaman *et al*., 2014; Roux *et al*., 2014; Roux *et al*., 2016). Additionally, S^0^ has been observed to be introduced into OMZs through the drifting off of S^0^ produced in coastal waters experiencing sulfidic events (Callbeck *et al*., 2018). These findings highlight the importance of considering full models of the sulfur cycle and avoiding simplified two‐reaction representations consisting only of dissimilatory sulfate reduction and chemolithotrophic re‐oxidation of sulfide to sulfate (Ulloa *et al*., 2012; Hawley *et al*., 2014). The extent of the other fluxes is currently a major unknown factor in DMW, with a large impact on the routes of sulfur‐driven carbon fixation and on the occurrence of other linkages with the carbon and nitrogen cycles.

### Sulfur‐reducing bacteria and Marinimicrobia

The consumption of SCIs in DMW is thought to proceed through a combination of oxidation, reduction and disproportionation (Sorokin *et al*., 1995; Zopfi *et al*., 2001; Sørensen and Canfield, 2004). The relative importance of these consumption routes is currently unknown. Sulfur isotope fractionation studies offer little insight, since the measurements in various euxinic marine waters can be explained by sulfate reduction as well as sulfur reduction or disproportionation (Li *et al*., 2010; Kamyshny *et al*., 2011). The reduction or disproportionation of S^0^ and thiosulfate is more exergonic than sulfate reduction under the conditions found in DMW (Supporting Information Methods, Table S3), implying that SRB could gain more energy through these reactions. Many cultured SRB are able to reduce or disproportionate thiosulfate (Rabus *et al*., 2015) through the Dsr pathway and thiosulfate reductase (PhsABC; Fig. 2; Burns and DiChristina, 2009) and may prefer this electron acceptor over sulfate (Jørgensen, 1990). Many anaerobic microorganisms use S^0^ as electron acceptor (Rabus *et al*., 2013) mediated by polysulfide reductase (PsrABC) or sulfur reductase (SreABC; Fig. 2; Laska *et al*., 2003; Sorokin *et al*., 2015). These three protein complexes (Phs, Psr, Sre) are complex iron–sulfur molybdoenzymes with such a close phylogenetic relationship and with so few characterized representatives, that distinction based on sequence is currently impossible (Hedderich *et al*., 1998; Hinsley and Berks, 2002; Laska *et al*., 2003; Duval *et al*., 2008; Burns and DiChristina, 2009). Furthermore, various SOB also encode genes with similarity to *psrABC* (Wright *et al*., 2014), of which the resulting enzymes may well act in reverse (Eddie and Hanson, 2013; Weissgerber *et al*., 2013). Thus, the presence of *psr‐*like genes in a genome suggests the capability of some form of dissimilatory sulfur conversion, but this requires further investigation.

The reduction of SCIs in DMW was used as energy metabolism by an organoheterotrophic *Shewanella* strain isolated from the Black Sea (Perry *et al*., 1993). However, related microorganisms are unlikely to play a big role in DMW, as they have not been detected in microbial ecology studies. SRB remain probable candidates for mediating sulfur reduction, as several genomes of putative SRB retrieved from the Black Sea encoded *psr*‐like genes and a tetrathionate reductase gene (*otr*) in addition to their sulfate‐reducing genes (Fig. 5). Other MAGs from DMW metagenomes also showed possibly reductive *psr*‐like genes, such as *Bacteroidia* NIOZ‐UU65 from the Black Sea, a MAG of uncultivated clade SAR324 from the Arabian Sea OMZ core and three *Marinimicrobia* genomes (Fig. 5). Bacteria of the uncultivated candidate phylum *Marinimicrobia* (formerly known as Marine Group A and clade SAR406) are prevalent in DMW and contain genomic signatures of organoheterotrophy (Wright *et al*., 2014; Bertagnolli *et al*., 2017; Hawley *et al*., 2017), a metabolism supported by DNA‐based stable isotope probing incubations from the Black Sea (Suominen *et al*., 2019). It has been suggested that *Marinimicrobia* may reduce S^0^ based on the presence of *psrABC* genes (Wright *et al*., 2014; Hawley *et al*., 2017). As explained, we think it would be more accurate and unambiguous to broaden the hypothesis to *Marinimicrobia* having an unspecified dissimilatory sulfur metabolism. Furthermore, the preference for shallow waters with relatively oxidizing conditions by *Marinimicrobia* NIOZ‐UU73 (Fig. 3A; 90% complete, no contamination) suggests that for this specific member, a facultative sulfur‐oxidizing lifestyle is more likely. Another *Marinimicrobia* MAG (PN262000N21, 98% complete, no contamination) encodes an almost‐complete ‘Sox’ pathway conferring the potential for dissimilatory thiosulfate oxidation (Figs 2 and 5). The most straightforward path to revealing the energy metabolism of these uncultivated bacteria would be cultivation, isolation and characterization. Like genomes of SAR11 and SUP05 bacteria, *Marinimicrobia* genomes are extensively streamlined (Hawley *et al*., 2017) implying that these bacteria are highly adapted to *in situ* conditions. Hence, cultivation may require natural seawater as medium, or recently designed synthetic alternatives (Henson *et al*., 2016).

### Sulfur‐disproportionating bacteria

Sulfur disproportionation is the simultaneous oxidation and reduction of an SCI, typically leading to the production of both sulfate and sulfide, which is an uncommon microbial trait (Finster, 2008; Slobodkin and Slobodkina, 2019). The biochemistry of sulfur disproportionation is unresolved and may involve the Dsr pathway in *Deltaproteobacteria* such as *Desulfurivibrio alkaliphilus* (Thorup *et al*., 2017) and *Desulfocapsa sulfexigens* (Finster, 2008; Finster *et al*., 2013), and Psr‐like molybdoenzymes and rhodanese sulfurtransferases in other microorganisms such as *Desulfurella amilsii* (Florentino *et al*., 2019). Although uncommon, disproportionation is probably influential in euxinic marine waters, as microorganisms growing through disproportionation of S^0^ or thiosulfate could be cultivated from the euxinic Mariager Fjord (Sørensen and Canfield, 2004), and a diffusion–reaction model of Chesapeake Bay required the inclusion of S^0^ disproportionation or reduction to explain the observed S^0^ concentration profiles (Findlay *et al*., 2017). In euxinic basins *Desulfocapsa* species could be involved, as their 16S rRNA genes were detected in the Cariaco Basin (Rodriguez‐Mora *et al*., 2015) as well as the Black Sea (Neretin *et al*., 2007; Fuchsman *et al*., 2011; Fuchsman *et al*., 2012). This hypothesis is difficult to test with genomic data due to the unclear biochemistry behind S^0^ disproportionation. Out of the MAGs obtained from the Black Sea, ^*U*^ *Df. sulfidica* is the most closely related to *Desulfocapsa*. Yet, it has a markedly different gene repertoire than *Dc. sulfexigens*, without molybdoenzymes or high numbers of rhodanese genes. However, plenty other sulfur‐disproportionating bacterial candidates with Dsr pathways, molybdoenzymes and high numbers of rhodanese genes remain (Fig. 5). Like *Dv. alkaliphilus* (Thorup *et al*., 2017), some might oxidize sulfide in a disproportionation‐dependent pathway including sulfide oxidation by Sqr (^*U*^ *Db. maris*, ^*U*^ *Db. vada*, ^*U*^ *Dl. nitratireducens*, Arabian Sea OMZ core *Desulfobacterales*).

Since most characterized sulfur‐disproportionating microorganisms can grow autotrophically (Finster *et al*., 1998; Florentino *et al*., 2016; Mardanov *et al*., 2016; Slobodkin and Slobodkina, 2019), their presence could spell a role in the high rates of carbon fixation that are generally observed within euxinic marine waters just below the euxinic interface (Jørgensen *et al*., 1991; Taylor *et al*., 2001). This phenomenon is commonly attributed solely to sulfur oxidation by chemolithoautotrophic SOB (Grote *et al*., 2008; Glaubitz *et al*., 2010). Our hypothesis is in agreement with the stimulation of carbon fixation measured upon the addition of thiosulfate or polysulfide to euxinic samples from the Baltic Sea (Labrenz *et al*., 2005; Jost *et al*., 2010). However, these findings could be influenced by artificial introduction of oxygen (De Brabandere *et al*., 2012) and are in need of further testing. In general, more dedicated experimental work is needed to quantitatively constrain SCI‐consuming reactions, such as was done for a freshwater lake (Findlay and Kamyshny, 2017). Another unexplored factor in the marine sulfur cycle is the cycling of organic sulfur compounds, which has been highlighted for marine sediments (Wasmund *et al*., 2017). Such processes may be important in DMW, as dimethylsulfide oxidation genes (*ddhA*) were detected in genomes of SUP05 bacteria, ^*U*^ *T. autotrophica* and *Marinimicrobia*, and dimethylsulfoxide reductase genes (*dmsA*) in MAGs of *Desulfacyla* species, ^*U*^ *Db. maris* and OMZ *Desulfobacterales*. Future studies should address to what extent reactions of SCIs and organic sulfur compounds contribute to the overall sulfur cycle, and whether this is affected by environmental conditions.

## Conclusions and future perspectives

**Fig 6:**
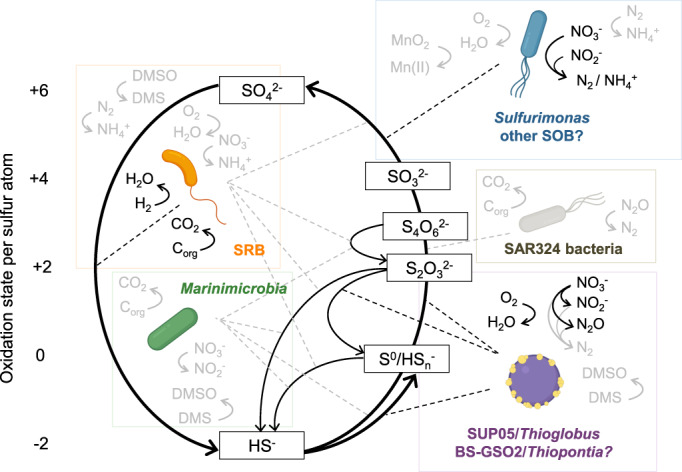
Conceptual ecophysiological model of the sulfur cycle and the involved microorganisms in DMW. Question marks and the light gray colour are used when there are indications for involvement of specific microorganisms and/or processes but definitive proof is lacking. Bacterial images were created with BioRender. [Color figure can be viewed at wileyonlinelibrary.com]

This review has presented a compendium of the current insights into sulfur‐cycling bacteria in DMW (Fig. 6). Based on current experimental evidence, it is difficult to investigate *in situ* sulfur reactions beyond sulfide oxidation and sulfate reduction. Euxinic marine waters are thought to host a complex network of reactions, whereas this remains more uncertain for suboxic waters and OMZ cores. Molecular studies have revealed a high diversity of putative SRB and SOB, which we expect to be explored further and consolidated over the coming years, specifically with the use of improved genome‐centric metagenomics. Sampling methods without bias against particle‐associated microorganisms can give an accurate and intercomparable view on diversity and abundance across DMW, specifically of SRB. With this in mind, it could be evaluated whether the community of novel putative SRB genomically revealed by us in the Black Sea is representative of euxinic marine basins and perhaps DMW in general. Together, the diverse sulfur‐cycling bacteria form a myriad of connections with other elemental cycles. SUP05 bacteria fix inorganic carbon with energy from very low sulfide concentrations, warranting a biologically meaningful reshaping of our concept of euxinia. These insights into sulfide affinity are crucial building blocks for biogeochemical modelling efforts, which could be further improved by estimations of critical biological parameters including biomass yield and sulfide tolerance. Metatranscriptomics and metaproteomics experiments might play a role in testing under which conditions SRB use their genomic potential for respiration of oxygen and diverse nitrogen compounds. Notably, genomic classifications of some bacteria as ‘SRB’ are for now putative, as the Dsr pathway on which this is based could also confer other forms of sulfur metabolism, such as sulfur reduction, disproportionation or oxidation. Similarly, the lack of fundamental insight into the relation of sequence and function of prevalent Psr‐related molybdoenzymes hinders metabolic predictions. Thus, genomic‐based research can find strong support in cultivation experiments and the *in vitro* study of heterologously expressed sulfur enzymes. The power of cultivation has been showcased by the isolation of SUP05, *Sulfurimonas* and *Arcobacter* bacteria, and specifically that of the Mn‐reducing and probably nitrogen‐fixing ‘*Ca. S. marisnigri*’. The cultivation and isolation of SRB from DMW is also feasible (Teske *et al*., 1996; Zopfi *et al*., 2001; Sørensen and Canfield, 2004; Finster and Kjeldsen, 2010), but more challenging than cultivation from sediment due to rapid oxidation of sampled water. These efforts could be facilitated by genome‐guided cultivation (Gutleben *et al*., 2018) or a reverse genomic approach (Cross *et al*., 2019). Finally, the factors controlling nitrogen fixation by SOB and SRB require further investigation, as this may be an important factor in the expected development of open‐ocean euxinia (Ulloa *et al*., 2012). The advances as summarized and predicted herein will enable the construction of biogeochemical models of the sulfur cycle from meta‐omics data, as has been done for the nitrogen cycle in the Arabian Sea (Reed *et al*., 2014) and in the Saanich Inlet (Louca *et al*., 2016). In the future, such endeavors could aid in predicting the biogeochemical response to expanding dysoxia and euxinia.

## Conflict of interest

The authors declare no conflict of interest.

## Supporting information

**Data S1.** Maximum‐likelihood phylogenetic reconstruction of *dsrA* genes in newick format. See Supporting Information Methods for methodology.

**Table S1.** Microbiological and biogeochemical studies of the sulfur cycle in dysoxic marine waters, grouped by environment. Volumes based on the work of Paulmier and Ruiz‐Pino (2009) correspond to the estimated volume of waters containing > 0.5 μM nitrite. The maximum volume of anoxic water off the Namibian coast was calculated from the largest observed extent of sulfidic bottom waters (7000 km^2^; Lavik *et al*., 2009) and an assumed sulfidic layer thickness of 10 m.

**Table S2.** Origin, quality, classification, annotation, and average amino acid identity (AAI) of the analysed genomes in Fig. 5. Methods are described in Supporting Information Methods. The AAI values were calculated with an *enveomics* script using Diamond because of computational limitations, which could lead to significantly overestimated AAI values between 50% and 60% (https://rodriguez-r.com/blog/aai-blast-vs-diamond/). Following the thresholds proposed by Konstantinidis and colleagues (2017), values exceeding the 65% genus‐level lower threshold are coloured green, and values exceeding the 45% family‐level lower threshold are coloured yellow.

**Table S3.** Gibbs free energies [ΔG (kJ e^−^)] of common dissimilatory conversions mediated by anaerobic microorganisms under conditions representative of the upper euxinic zone of the Black Sea and the core of the ETSP OMZ. Calculation methodology and variables used can be found in Supporting Information Methods.

**Appendix S1.** Supplementary Information Methods.

**Appendix S2.** Supplementary Information Protologue.
