# Supplementary material for: The bacterial sulfur cycle in expanding dysoxic and euxinic marine waters
Source: Environ Microbiol. 2020 Oct 18;23(6):2834–57. doi: 10.1111/1462-2920.15265 (PMC8359478; doi:10.1111/1462-2920.15265)
Supplement: Supplementary file 2 — Table S1. Microbiological and biogeochemical studies of the sulfur cycle in dysoxic marine waters, grouped by environment. Volumes based on the work of Paulmier and Ruiz‐Pino (2009) correspond to the estimated volume of waters containing > 0.5 μM nitrite. The maximum volume of anoxic water off the Namibian coast was calculated from the largest observed extent of sulfidic bottom waters (7000 km2; Lavik et al., 2009) and an assumed sulfidic layer thickness of 10 m. [file EMI-23-2834-s003.docx]

**Table S1.** Microbiological and biogeochemical studies of the sulfur cycle in dysoxic marine waters, grouped by environment. Volumes based on the work of Paulmier and Ruiz-Pino (2009) correspond to the estimated volume of waters containing >0.5 µM nitrite. The maximum volume of anoxic water off the Namibian coast was calculated from the largest observed extent of sulfidic bottom waters (7,000 km^2^; Lavik et al., 2009) and an assumed sulfidic layer thickness of 10 m.

| Location | Environment | Volume (m^3^) | Studies |
| --- | --- | --- | --- |
| Arabian Sea | OMZ core | 4.6 · 10^14^ ([Paulmier and Ruiz-Pino, 2009](#_ENREF_47)) | ([Reed et al., 2014](#_ENREF_52)) |
| Eastern tropical North Pacific (ETNP) | OMZ core | 2 · 10^15^ ([Paulmier and Ruiz-Pino, 2009](#_ENREF_47)) | ([Wright et al., 2014](#_ENREF_72); [Carolan et al., 2015](#_ENREF_10); [Plominsky et al., 2018](#_ENREF_51)) |
| Eastern tropical South Pacific (ETSP), Peru and Chile coastal shelf not included | OMZ core | 2.4 · 10^13^ – 2 · 10^14^ ([Thamdrup et al., 2012](#_ENREF_67)) | ([Canfield et al., 2010](#_ENREF_9); [Finster and Kjeldsen, 2010](#_ENREF_16); [Stewart et al., 2012](#_ENREF_62); [Ulloa et al., 2012](#_ENREF_69); [Azhar et al., 2014](#_ENREF_3); [Ganesh et al., 2014](#_ENREF_17); [Callbeck et al., 2018](#_ENREF_8); [Martínez-Pérez et al., 2018](#_ENREF_40); [Plominsky et al., 2018](#_ENREF_51)) |
| Baltic Sea (Gdansk Deep, Gotland Deep and Landsort Deep) | Basin | <1.6 · 10^10^ ([Hansson et al., 2011](#_ENREF_22)) | ([Labrenz et al., 2005](#_ENREF_32); [Brettar et al., 2006](#_ENREF_5); [Glaubitz et al., 2008](#_ENREF_20); [Grote et al., 2008](#_ENREF_21); [Jost et al., 2010](#_ENREF_28); [Glaubitz et al., 2013](#_ENREF_19); [Kamyshny et al., 2013](#_ENREF_29); [Labrenz et al., 2013](#_ENREF_33); [Korneeva et al., 2015](#_ENREF_31); [Rogge et al., 2017](#_ENREF_54); [Möller et al., 2019](#_ENREF_42)) |
| Black Sea | Basin | 4.7 · 10^14^ ([Sorokin, 2002](#_ENREF_60)) | ([Sorokin, 1972](#_ENREF_59); [Murray et al., 1989](#_ENREF_44); [Jørgensen et al., 1991](#_ENREF_27); [Overmann et al., 1992](#_ENREF_46); [Albert et al., 1995](#_ENREF_1); [Sorokin et al., 1995](#_ENREF_61); [Pimenov et al., 2000](#_ENREF_48); [Sorokin, 2002](#_ENREF_60); [Manske et al., 2005](#_ENREF_38); [Konovalov et al., 2006](#_ENREF_30); [Neretin et al., 2007](#_ENREF_45); [Yakushev et al., 2007](#_ENREF_73); [Grote et al., 2008](#_ENREF_21); [Glaubitz et al., 2010](#_ENREF_18); [Marschall et al., 2010](#_ENREF_39); [Glaubitz et al., 2013](#_ENREF_19); [Azhar et al., 2014](#_ENREF_3); [Henkel et al., 2019](#_ENREF_25)) |
| Cariaco Basin | Basin | 5.2 · 10^12^ ([Taylor et al., 2001](#_ENREF_65)) | ([Taylor et al., 2001](#_ENREF_65); [Li et al., 2008](#_ENREF_36); [Wakeham et al., 2012](#_ENREF_70); [Rodriguez-Mora et al., 2016](#_ENREF_53); [Suter et al., 2018](#_ENREF_63); [Taylor et al., 2018](#_ENREF_64)) |
| Marine Lake Rogoznica | Basin | 8.6 · 10^4^ ([Mihelčić et al., 1996](#_ENREF_41)) | ([Pjevac et al., 2015](#_ENREF_49); [Pjevac et al., 2019](#_ENREF_50)) |
| Chesapeake Bay | Coastal | <4.8 · 10^7^ ([Lewis et al., 2007](#_ENREF_35)) | ([Findlay et al., 2014](#_ENREF_15); [Findlay et al., 2015](#_ENREF_14); [Findlay et al., 2017](#_ENREF_13)) |
| Namibian Upwelling Shelf | Coastal | <7 · 10^10^ ([Lavik et al., 2009](#_ENREF_34)) | ([Brüchert et al., 2003](#_ENREF_6); [Lavik et al., 2009](#_ENREF_34)) |
| Peru and Chile Coastal Shelf | Coastal | 1.3 · 10^14^ ([Paulmier and Ruiz-Pino, 2009](#_ENREF_47)) | ([Schunck et al., 2013](#_ENREF_55); [Murillo et al., 2014](#_ENREF_43); [Hawley et al., 2017](#_ENREF_24); [Callbeck et al., 2018](#_ENREF_8); [Crowe et al., 2018](#_ENREF_11); [Plominsky et al., 2018](#_ENREF_51); [Callbeck et al., 2019](#_ENREF_7)) |
| Effingham Inlet | Fjord/inlet | <1.9 · 10^8^ ([Ingall et al., 2005](#_ENREF_26)) | ([Shah et al., 2017](#_ENREF_56); [Shah et al., 2019](#_ENREF_57)) |
| Golfo Dulce | Fjord/inlet | <2.5 · 10^9^ ([Ferdelman et al., 2006](#_ENREF_12)) | ([Thamdrup et al., 1996](#_ENREF_68); [Ferdelman et al., 2006](#_ENREF_12); [Bertagnolli et al., 2017](#_ENREF_4)) |
| Mariager Fjord | Fjord/Inlet | <1.2 · 10^8^ ([Zopfi et al., 2001](#_ENREF_74)) | ([Teske et al., 1996](#_ENREF_66); [Zopfi et al., 2001](#_ENREF_74); [Sørensen and Canfield, 2004](#_ENREF_58)) |
| Saanich Inlet | Fjord/inlet | <2 · 10^9^ ([Anderson and Devol, 1973](#_ENREF_2)) | ([Anderson and Devol, 1973](#_ENREF_2); [Walsh et al., 2009](#_ENREF_71); [Hawley et al., 2014](#_ENREF_23); [Wright et al., 2014](#_ENREF_72); [Louca et al., 2016](#_ENREF_37); [Hawley et al., 2017](#_ENREF_24)) |

# References

Albert, D.B., Taylor, C., and Martens, C.S. (1995) Sulfate reduction rates and low-molecular-weight fatty-acid concentrations in the water column and surficial sediments of the Black Sea. *Deep Sea Res Part I Oceanogr Res Pap* **42**: 1239-1260.

Anderson, J.J., and Devol, A.H. (1973) Deep water renewal in Saanich Inlet, an intermittently anoxic basin. *Estuarine and Coastal Marine Science* **1**: 1-10.

Azhar, M.A., Canfield, D.E., Fennel, K., Thamdrup, B., and Bjerrum, C.J. (2014) A model-based insight into the coupling of nitrogen and sulfur cycles in a coastal upwelling system. *Journal of Geophysical Research: Biogeosciences* **119**: 264-285.

Bertagnolli, A.D., Padilla, C.C., Glass, J.B., Thamdrup, B., and Stewart, F.J. (2017) Metabolic potential and in situ activity of marine *Marinimicrobia* bacteria in an anoxic water column. *Environ Microbiol* **19**: 4392-4416.

Brettar, I., Labrenz, M., Flavier, S., Botel, J., Kuosa, H., Christen, R., and Hofle, M.G. (2006) Identification of a *Thiomicrospira denitrificans*-like epsilonproteobacterium as a catalyst for autotrophic denitrification in the central Baltic Sea. *Appl Environ Microbiol* **72**: 1364-1372.

Brüchert, V., Jørgensen, B.B., Neumann, K., Riechmann, D., Schlösser, M., and Schulz, H. (2003) Regulation of bacterial sulfate reduction and hydrogen sulfide fluxes in the central Namibian coastal upwelling zone. *Geochim Cosmochim Acta* **67**: 4505-4518.

Callbeck, C.M., Pelzer, C., Lavik, G., Ferdelman, T.G., Graf, J.S., Vekeman, B. et al. (2019) *Arcobacter peruensis* sp. nov., a chemolithoheterotroph isolated from sulfide-and organic-rich coastal waters off Peru. *Applied Environ Microbiol* **85**.

Callbeck, C.M., Lavik, G., Ferdelman, T.G., Fuchs, B., Gruber-Vodicka, H.R., Hach, P.F. et al. (2018) Oxygen minimum zone cryptic sulfur cycling sustained by offshore transport of key sulfur oxidizing bacteria. *Nat Commun* **9**: 1729.

Canfield, D.E., Stewart, F.J., Thamdrup, B., De Brabandere, L., Dalsgaard, T., Delong, E.F. et al. (2010) A cryptic sulfur cycle in oxygen-minimum-zone waters off the Chilean coast. *Science* **330**: 1375-1378.

Carolan, M.T., Smith, J.M., and Beman, J.M. (2015) Transcriptomic evidence for microbial sulfur cycling in the eastern tropical North Pacific oxygen minimum zone. *Front Microbiol* **6**: 334.

Crowe, S.A., Cox, R.P., Jones, C., Fowle, D.A., Santibanez-Bustos, J.F., Ulloa, O., and Canfield, D.E. (2018) Decrypting the sulfur cycle in oceanic oxygen minimum zones. *ISME J* **12**: 2322-2329.

Ferdelman, T.G., Thamdrup, B., Canfield, D.E., Glud, R.N., Kuever, J., Lillebæk, R. et al. (2006) Biogeochemical controls on the oxygen, nitrogen and sulfur distributions in the water column of Golfo Dulce: an anoxic basin on the Pacific coast of Costa Rica revisited. *Rev Biol Trop* **54**: 171-191.

Findlay, A.J., Di Toro, D.M., and Luther, G.W. (2017) A model of phototrophic sulfide oxidation in a stratified estuary. *Limnol Oceanogr* **62**: 1853-1867.

Findlay, A.J., Bennett, A.J., Hanson, T.E., and Luther, G.W., 3rd (2015) Light-dependent sulfide oxidation in the anoxic zone of the Chesapeake Bay can be explained by small populations of phototrophic bacteria. *Appl Environ Microbiol* **81**: 7560-7569.

Findlay, A.J., Gartman, A., MacDonald, D.J., Hanson, T.E., Shaw, T.J., and Luther, G.W. (2014) Distribution and size fractionation of elemental sulfur in aqueous environments: The Chesapeake Bay and Mid-Atlantic Ridge. *Geochim Cosmochim Acta* **142**: 334-348.

Finster, K.W., and Kjeldsen, K.U. (2010) *Desulfovibrio oceani* subsp. *oceani* sp. nov., subsp. nov. and *Desulfovibrio oceani* subsp. *galateae* subsp. nov., novel sulfate-reducing bacteria isolated from the oxygen minimum zone off the coast of Peru. *Antonie Van Leeuwenhoek* **97**: 221-229.

Ganesh, S., Parris, D.J., DeLong, E.F., and Stewart, F.J. (2014) Metagenomic analysis of size-fractionated picoplankton in a marine oxygen minimum zone. *ISME J* **8**: 187-211.

Glaubitz, S., Labrenz, M., Jost, G., and Jurgens, K. (2010) Diversity of active chemolithoautotrophic prokaryotes in the sulfidic zone of a Black Sea pelagic redoxcline as determined by rRNA-based stable isotope probing. *FEMS Microbiol Ecol* **74**: 32-41.

Glaubitz, S., Kiesslich, K., Meeske, C., Labrenz, M., and Jurgens, K. (2013) SUP05 dominates the Gammaproteobacterial sulfur oxidizer assemblages in pelagic redoxclines of the central Baltic and Black Seas. *Appl Environ Microbiol* **79**: 2767-2776.

Glaubitz, S., Lueders, T., Abraham, W.R., Jost, G., Jürgens, K., and Labrenz, M. (2008) 13C‐isotope analyses reveal that chemolithoautotrophic *Gamma*‐and *Epsilonproteobacteria* feed a microbial food web in a pelagic redoxcline of the central Baltic Sea. *Environ Microbiol* **11**: 326-337.

Grote, J., Jost, G., Labrenz, M., Herndl, G.J., and Jurgens, K. (2008) *Epsilonproteobacteria* represent the major portion of chemoautotrophic bacteria in sulfidic waters of pelagic redoxclines of the Baltic and Black Seas. *Appl Environ Microbiol* **74**: 7546-7551.

Hansson, M., Andersson, L., and Axe, P. (2011) *Areal Extent and Volume of Anoxia and Hypnoxia in the Baltic Sea, 1960-2011*: SMHI.

Hawley, A.K., Brewer, H.M., Norbeck, A.D., Pasa-Tolic, L., and Hallam, S.J. (2014) Metaproteomics reveals differential modes of metabolic coupling among ubiquitous oxygen minimum zone microbes. *Proc Natl Acad Sci USA* **111**: 11395-11400.

Hawley, A.K., Nobu, M.K., Wright, J.J., Durno, W.E., Morgan-Lang, C., Sage, B. et al. (2017) Diverse *Marinimicrobia* bacteria may mediate coupled biogeochemical cycles along eco-thermodynamic gradients. *Nat Commun* **8**: 1507.

Henkel, J.V., Dellwig, O., Pollehne, F., Herlemann, D.P.R., Leipe, T., and Schulz-Vogt, H.N. (2019) A bacterial isolate from the Black Sea oxidizes sulfide with manganese(IV) oxide. *Proc Natl Acad Sci U S A*: 201906000.

Ingall, E., Kolowith, L., Lyons, T., and Hurtgen, M. (2005) Sediment carbon, nitrogen and phosphorus cycling in an anoxic fjord, Effingham Inlet, British Columbia. *Am J Sci* **305**: 240-258.

Jørgensen, B.B., Fossing, H., Wirsen, C.O., and Jannasch, H.W. (1991) Sulfide oxidation in the anoxic Black Sea chemocline. *Deep Sea Research Part A Oceanographic Research Papers* **38**: S1083-S1103.

Jost, G., Martens-Habbena, W., Pollehne, F., Schnetger, B., and Labrenz, M. (2010) Anaerobic sulfur oxidation in the absence of nitrate dominates microbial chemoautotrophy beneath the pelagic chemocline of the eastern Gotland Basin, Baltic Sea. *FEMS Microbiol Ecol* **71**: 226-236.

Kamyshny, A., Yakushev, E.V., Jost, G., and Podymov, O.I. (2013) Role of sulfide oxidation intermediates in the redox balance of the oxic–anoxic interface of the Gotland Deep, Baltic Sea. In *Chemical Structure of Pelagic Redox Interfaces: Observation and Modeling*. Berlin/Heidelberg, Germany: Springer, pp. 95-119.

Konovalov, S.K., Murray, J.W., Luther, G.W., and Tebo, B.M. (2006) Processes controlling the redox budget for the oxic/anoxic water column of the Black Sea. *Deep Sea Res Part II Top Stud Oceanogr* **53**: 1817-1841.

Korneeva, V.A., Pimenov, N.V., Krek, A.V., Tourova, T.P., and Bryukhanov, A.L. (2015) Sulfate-reducing bacterial communities in the water column of the Gdansk Deep (Baltic Sea). *Mikrobiologiia* **84**: 250-260.

Labrenz, M., Jost, G., Pohl, C., Beckmann, S., Martens-Habbena, W., and Jurgens, K. (2005) Impact of different in vitro electron donor/acceptor conditions on potential chemolithoautotrophic communities from marine pelagic redoxclines. *Appl Environ Microbiol* **71**: 6664-6672.

Labrenz, M., Grote, J., Mammitzsch, K., Boschker, H.T., Laue, M., Jost, G. et al. (2013) *Sulfurimonas gotlandica* sp. nov., a chemoautotrophic and psychrotolerant epsilonproteobacterium isolated from a pelagic redoxcline, and an emended description of the genus *Sulfurimonas*. *Int J Syst Evol Microbiol* **63**: 4141-4148.

Lavik, G., Stuhrmann, T., Bruchert, V., Van der Plas, A., Mohrholz, V., Lam, P. et al. (2009) Detoxification of sulphidic African shelf waters by blooming chemolithotrophs. *Nature* **457**: 581-584.

Lewis, B.L., Glazer, B.T., Montbriand, P.J., Luther III, G.W., Nuzzio, D.B., Deering, T. et al. (2007) Short-term and interannual variability of redox-sensitive chemical parameters in hypoxic/anoxic bottom waters of the Chesapeake Bay. *Mar Chem* **105**: 296-308.

Li, X.N., Taylor, G.T., Astor, Y., and Scranton, M.I. (2008) Relationship of sulfur speciation to hydrographic conditions and chemoautotrophic production in the Cariaco Basin. *Mar Chem* **112**: 53-64.

Louca, S., Hawley, A.K., Katsev, S., Torres-Beltran, M., Bhatia, M.P., Kheirandish, S. et al. (2016) Integrating biogeochemistry with multiomic sequence information in a model oxygen minimum zone. *Proc Natl Acad Sci USA* **113**: E5925-E5933.

Manske, A.K., Glaeser, J., Kuypers, M.M., and Overmann, J. (2005) Physiology and phylogeny of green sulfur bacteria forming a monospecific phototrophic assemblage at a depth of 100 meters in the Black Sea. *Appl Environ Microbiol* **71**: 8049-8060.

Marschall, E., Jogler, M., Hessge, U., and Overmann, J. (2010) Large-scale distribution and activity patterns of an extremely low-light-adapted population of green sulfur bacteria in the Black Sea. *Environ Microbiol* **12**: 1348-1362.

Martínez-Pérez, C., Mohr, W., Schwedt, A., Dürschlag, J., Callbeck, C.M., Schunck, H. et al. (2018) Metabolic versatility of a novel N_2_-fixing *Alphaproteobacterium* isolated from a marine oxygen minimum zone. *Environ Microbiol* **20**: 755-768.

Mihelčić, G., Šurija, B., Juračić, M., Barišić, D., and Branica, M. (1996) History of the accumulation of trace metals in sediments of the saline Rogoznica Lake (Croatia). *Sci Total Environ* **182**: 105-115.

Möller, L., Laas, P., Rogge, A., Goetz, F., Bahlo, R., Leipe, T., and Labrenz, M. (2019) *Sulfurimonas* subgroup GD17 cells accumulate polyphosphate under fluctuating redox conditions in the Baltic Sea: possible implications for their ecology. *ISME J* **13**: 482-493.

Murillo, A.A., Ramírez-Flandes, S., DeLong, E.F., and Ulloa, O. (2014) Enhanced metabolic versatility of planktonic sulfur-oxidizing *γ-proteobacteria* in an oxygen-deficient coastal ecosystem. *Front Mar Sci* **1**: 18.

Murray, J.W., Jannasch, H.W., Honjo, S., Anderson, R.F., Reeburgh, W.S., Top, Z. et al. (1989) Unexpected changes in the oxic anoxic interface in the Black Sea. *Nature* **338**: 411-413.

Neretin, L.N., Abed, R.M., Schippers, A., Schubert, C.J., Kohls, K., and Kuypers, M.M. (2007) Inorganic carbon fixation by sulfate-reducing bacteria in the Black Sea water column. *Environ Microbiol* **9**: 3019-3024.

Overmann, J., Cypionka, H., and Pfennig, N. (1992) An extremely low-light-adapted phototrophic sulfur bacterium from the Black Sea. *Limnol Oceanogr* **37**: 150-155.

Paulmier, A., and Ruiz-Pino, D. (2009) Oxygen minimum zones (OMZs) in the modern ocean. *Prog Oceanogr* **80**: 113-128.

Pimenov, N.V., Rusanov, I.I., Yusupov, S.K., Fridrich, J., Lein, A.Y., Wehrli, B., and Ivanov, M.V. (2000) Microbial processes at the aerobic-anaerobic interface in the deep-water zone of the Black Sea. *Microbiology* **69**: 436-448.

Pjevac, P., Korlevic, M., Berg, J.S., Bura-Nakic, E., Ciglenecki, I., Amann, R., and Orlic, S. (2015) Community shift from phototrophic to chemotrophic sulfide oxidation following anoxic holomixis in a stratified seawater lake. *Appl Environ Microbiol* **81**: 298-308.

Pjevac, P., Dyksma, S., Goldhammer, T., Mujakić, I., Koblížek, M., Mussmann, M. et al. (2019) *In situ* abundance and carbon fixation activity of distinct anoxygenic phototrophs in the stratified seawater lake Rogoznica. *bioRxiv*: 631366.

Plominsky, A.M., Trefault, N., Podell, S., Blanton, J.M., De la Iglesia, R., Allen, E.E. et al. (2018) Metabolic potential and in situ transcriptomic profiles of previously uncharacterized key microbial groups involved in coupled carbon, nitrogen and sulfur cycling in anoxic marine zones. *Environ Microbiol* **20**: 2727-2742.

Reed, D.C., Algar, C.K., Huber, J.A., and Dick, G.J. (2014) Gene-centric approach to integrating environmental genomics and biogeochemical models. *Proc Natl Acad Sci USA* **111**: 1879-1884.

Rodriguez-Mora, M.J., Edgcomb, V.P., Taylor, C., Scranton, M.I., Taylor, G.T., and Chistoserdov, A.Y. (2016) The diversity of sulfide oxidation and sulfate reduction genes expressed by the bacterial communities of the Cariaco Basin, Venezuela. *Open Microbiol J* **10**: 140-149.

Rogge, A., Vogts, A., Voss, M., Jurgens, K., Jost, G., and Labrenz, M. (2017) Success of chemolithoautotrophic SUP05 and *Sulfurimonas* GD17 cells in pelagic Baltic Sea redox zones is facilitated by their lifestyles as K- and r-strategists. *Environ Microbiol* **19**: 2495-2506.

Schunck, H., Lavik, G., Desai, D.K., Grosskopf, T., Kalvelage, T., Loscher, C.R. et al. (2013) Giant hydrogen sulfide plume in the oxygen minimum zone off Peru supports chemolithoautotrophy. *PLoS One* **8**: e68661.

Shah, V., Chang, B.X., and Morris, R.M. (2017) Cultivation of a chemoautotroph from the SUP05 clade of marine bacteria that produces nitrite and consumes ammonium. *ISME J* **11**: 263–271.

Shah, V., Zhao, X., Lundeen, R.A., Ingalls, A.E., Nicastro, D., and Morris, R.M. (2019) Morphological plasticity in a sulfur-oxidizing marine bacterium from the SUP05 clade enhances dark carbon fixation. *mBio* **10**: e00216-00219.

Sørensen, K.B., and Canfield, D.E. (2004) Annual fluctuations in sulfur isotope fractionation in the water column of a euxinic marine basin. *Geochim Cosmochim Acta* **68**: 503-515.

Sorokin, Y.I. (1972) The bacterial population and the processes of hydrogen sulphide oxidation in the Black Sea. *Journal du Conseil* **34**: 423-454.

Sorokin, Y.I. (2002) *The Black Sea: ecology and oceanography*. Amsterdam, The Netherlands: Backhuys Publishers.

Sorokin, Y.I., Sorokin, P.Y., Avdeev, V.A., Sorokin, D.Y., and Ilchenko, S.V. (1995) Biomass, production and activity of bacteria in the Black Sea, with special reference to chemosynthesis and the sulfur cycle. *Hydrobiologia* **308**: 61-76.

Stewart, F.J., Ulloa, O., and DeLong, E.F. (2012) Microbial metatranscriptomics in a permanent marine oxygen minimum zone. *Environ Microbiol* **14**: 23-40.

Suter, E.A., Pachiadaki, M., Taylor, G.T., Astor, Y., and Edgcomb, V.P. (2018) Free-living chemoautotrophic and particle-attached heterotrophic prokaryotes dominate microbial assemblages along a pelagic redox gradient. *Environ Microbiol* **20**: 693-712.

Taylor, G.T., Suter, E.A., Pachiadaki, M.G., Astor, Y., Edgcomb, V.P., and Scranton, M.I. (2018) Temporal shifts in dominant sulfur-oxidizing chemoautotrophic populations across the Cariaco Basin's redoxcline. *Deep Sea Res Part II Top Stud Oceanogr* **156**: 80-96.

Taylor, G.T., Iabichella, M., Ho, T.Y., Scranton, M.I., Thunell, R.C., Muller-Karger, F., and Varela, R. (2001) Chemoautotrophy in the redox transition zone of the Cariaco Basin: A significant midwater source of organic carbon production. *Limnol Oceanogr* **46**: 148-163.

Teske, A., Wawer, C., Muyzer, G., and Ramsing, N.B. (1996) Distribution of sulfate-reducing bacteria in a stratified fjord (Mariager Fjord, Denmark) as evaluated by most-probable-number counts and denaturing gradient gel electrophoresis of PCR-amplified ribosomal DNA fragments. *Appl Environ Microbiol* **62**: 1405-1415.

Thamdrup, B., Dalsgaard, T., and Revsbech, N.P. (2012) Widespread functional anoxia in the oxygen minimum zone of the Eastern South Pacific. *Deep Sea Res Part I Oceanogr Res Pap* **65**: 36-45.

Thamdrup, B., Canfield, D.E., Ferdelman, T.G., Glud, R.N., and Gundersen, J.K. (1996) A biogeochemical survey of the anoxic basin Golfo Dulce, Costa Rica. *Rev Biol Trop*: 19-33.

Ulloa, O., Canfield, D.E., DeLong, E.F., Letelier, R.M., and Stewart, F.J. (2012) Microbial oceanography of anoxic oxygen minimum zones. *Proc Natl Acad Sci USA* **109**: 15996-16003.

Wakeham, S.G., Turich, C., Schubotz, F., Podlaska, A., Li, X.N.N., Varela, R. et al. (2012) Biomarkers, chemistry and microbiology show chemoautotrophy in a multilayer chemocline in the Cariaco Basin. *Deep Sea Res Part I Oceanogr Res Pap* **63**: 133-156.

Walsh, D.A., Zaikova, E., Howes, C.G., Song, Y.C., Wright, J.J., Tringe, S.G. et al. (2009) Metagenome of a versatile chemolithoautotroph from expanding oceanic dead zones. *Science* **326**: 578-582.

Wright, J.J., Mewis, K., Hanson, N.W., Konwar, K.M., Maas, K.R., and Hallam, S.J. (2014) Genomic properties of Marine Group A bacteria indicate a role in the marine sulfur cycle. *ISME J* **8**: 455-468.

Yakushev, E., Pollehne, F., Jost, G., Kuznetsov, I., Schneider, B., and Umlauf, L. (2007) Analysis of the water column oxic/anoxic interface in the Black and Baltic seas with a numerical model. *Mar Chem* **107**: 388-410.

Zopfi, J., Ferdelman, T.G., Jorgensen, B.B., Teske, A., and Thamdrup, B. (2001) Influence of water column dynamics on sulfide oxidation and other major biogeochemical processes in the chemocline of Mariager Fjord (Denmark). *Mar Chem* **74**: 29-51.
