# Supplementary material for: The bacterial sulfur cycle in expanding dysoxic and euxinic marine waters
Source: Environ Microbiol. 2020 Oct 18;23(6):2834–57. doi: 10.1111/1462-2920.15265 (PMC8359478; doi:10.1111/1462-2920.15265)
Supplement: Supplementary file 5 — Appendix S1. Supplementary Information Methods. [file EMI-23-2834-s005.docx]

# Supporting Information Methods

## Physicochemical data of the Black Sea

The methodology of the physicochemical measurements from sampling station 2 (N42°53.8’, E30°40.7, 2,107m depth) in the Black Sea western gyre during the Phoxy cruise 64PE371 (BS2013) on the 9th and 10th June 2013 on board the *R/V Pelagia* were described by Sollai et al. (2019). Redox potential was measured at the same station in 2016 on the 1^st^ and 2^nd^ of February 2016 during the 64PE408 ‘NESSC/SIAM’ cruise aboard the *R/V Pelagia*. Sampling was performed with a rosette sampler equipped with Go-Flow bottles (General Oceanics, Miami, FL, USA) and a conductivity-temperature-density (CTD) unit (SBE 911 plus, Sea-Bird Electronics, Bellevue, WA, USA). On deck, the bottles were pressurized with N_2_ to ensure anoxic conditions throughout the sampling procedure. Redox potential was measured with a ProSense QR400X-6MM Epoxy ORP redox electrode (Oosterhout, The Netherlands). Several hours were taken for equilibration, as is necessary for samples with neutral pH to have reproducible measurements (Boulegue and Michard, 1979). Sulfide was also measured from ZnCl_2_-complexed samples using the photometric methylene blue method (Cline, 1969), revealing a linear correlation with depth in the upper 500 m down to a concentration of 0.1 µM similar to previous reports (Jørgensen et al., 1991). Furthermore, the depth and potential density of the non-sulfidic/sulfidic interface was similar in all three profiles: 102 m in 2013, and 106 and 107 m in the two 2016 casts, corresponding to potential density values of 16.12, 16.16 and 16.13 kg m^-3^ respectively. Therefore, the physicochemical data of 2013 and the redox potential measurements of 2016 are combined in one plot in Figure 3A.


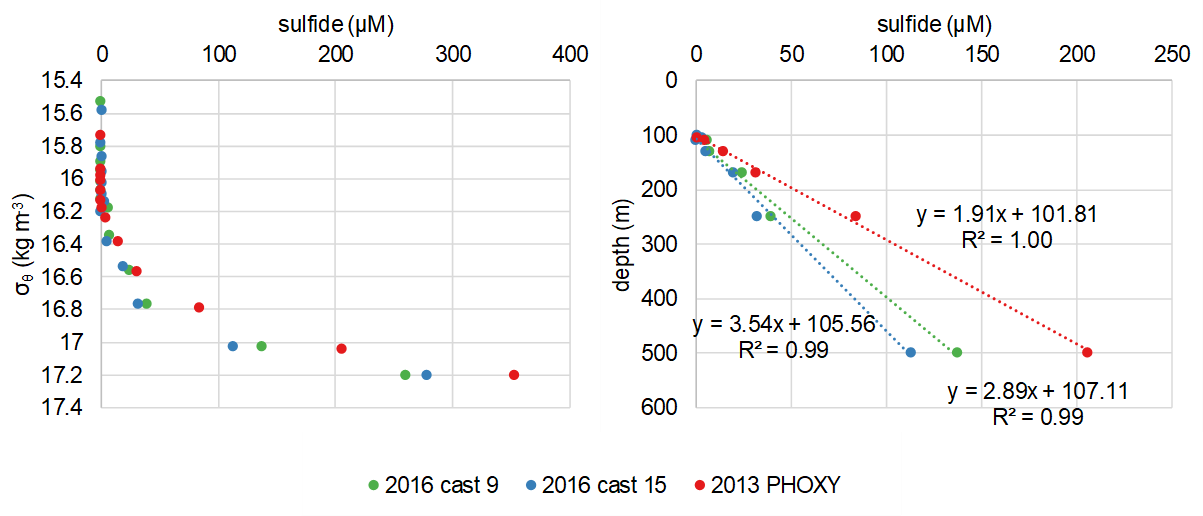


**Figure S1.** Sulfide measurements from Black Sea samples plotted against potential density (σ_θ_, left) and depth (right).

## Metagenomics and phylogenetic reconstruction

Suspended particulate matter (SPM) collection, sequencing, metagenome assembly and binning are described by Villanueva et al. (in press). In short, SPM was collected from 15 depths across the water column of sampling station 2 during the 2013 Phoxy cruise with *in-situ* pumps. Unamplified DNA extracts were sequenced with Illumina MiSeq, generating 45 million 2x250 bp paired-end reads. After quality control with FastQC v0.11.3^[[1]](#footnote-1)^ and trimming with Flexbar v2.5 (Dodt et al., 2012), reads were cross-assembled with metaSPAdes v3.8.0 (Bankevich et al., 2012) and mapped back to the assembly with BWA-MEM v0.7.12 (Li, 2013). The same methodology was applied to assemble ETSP OMZ metagenomes (Canfield et al., 2010; Ganesh et al., 2014). Scaffold read coverage profiles over different water column depths were based on the depth file generated during the binning step with the jgi_summarize_bam_contig_depths script. A biologically meaningful normalization of coverage was performed using single-copy genes (SCGs), similarly to for instance the normalization of read counts using the single-copy *rpoB* gene by Lüke et al. (2016). We analyzed the coverage of the 41 Pfam SCGs used by CheckM (Table S5) by querying the scaffolds as described in the following subsection with the Pfam HMM profiles (Finn et al., 2016) using the ‘trusted’ bitscore cutoff. The total coverage, i.e. the summed coverage across all sampling depths, was consistent for most SCGs (Figure S2). The coverage of SCGs at different water column water depths was averaged, and the average SCG coverage was used to normalize the coverage of scaffolds as follows:

$$normalized scaffold \mathrm{coverage}_{\mathrm{depth}}=\frac{\mathrm{scaffold}\mathrm{coverage}_{\mathrm{depth}}*average SCG coverage}{\mathrm{SCG}\mathrm{coverage}_{\mathrm{depth}}}$$


**Figure S2**. The residue length and total metagenome coverage of the 41 single-copy genes used for normalization.

Black Sea scaffolds were binned into metagenome-assembled genomes (MAGs) based on coverage profile across samples and tetra-nucleotide frequency with MetaBAT v0.32.4 (Kang et al., 2015) with the ‘—superspecific’ preset. Coverage of Black Sea MAGs was inferred from the normalized coverage of representative scaffolds encoding *dsr* or *sox* genes. In previous research, MAGs have been retrieved from marine metagenomes sampled during the TARA Oceans project (Parks et al., 2017; Tully et al., 2018). Based on physicochemical metadata, metagenomes and MAGs were subselected from the Arabian Sea OMZ core (TARA stations 37, 38, 39), the ETSP OMZ core (TARA stations 100, 102, 109, 110) and anoxic Mexican coastal waters (TARA station 111). The Black Sea and TARA MAGs were screened for dissimilatory sulfur genes as described in the following subsection. Additional genomes for phylogenetic reconstruction and functional annotation were selected from dysoxic marine water literature. Reference genomes were selected from GTDB r89 using AnnoTree v1.2.0 (Mendler et al., 2019). Quality of the genomes was assessed with CheckM v1.0.7 (Parks et al., 2015) in '--lineage_wf' mode. Black Sea MAGs were taxonomically classified with BAT (von Meijenfeldt et al., 2019) with the ‘f’ parameter set to 0.2, using the NCBI nr database dated January 8, 2019 and the dependencies Prodigal v2.6.3 (Hyatt et al., 2010) and Diamond v.0.9.21 (Buchfink et al., 2014). Additionally, all genomes were classified with GTDB-Tk v1.0.2 (Chaumeil et al., 2019) using GTDB r89 (Parks et al., 2017). They were screened for 16S rRNA genes with the ‘ssu_finder’ utility of CheckM. For phylogenetic reconstruction, genomes were selected based on >50% completeness, <10% contamination. A trimmed concatenated alignment of 120 single-copy household genes was constructed with the ‘–identify’ and ‘–align’ functionalities of GTDB-Tk. This alignment was used to construct a phylogenetic tree with IQ-TREE v1.6.10 (Nguyen et al., 2015) using the LG+C40+F+R4 evolutionary model determined to be the most suitable by ModelFinder based on the Akaike information criterion (Kalyaanamoorthy et al., 2017). Branch support was determined based on 1,000 bootstraps calculated by UFBoot2 (Hoang et al., 2017). Trees were inspected with FigTree v1.4.2^[[2]](#footnote-2)^ and visualized with iTOL (Letunic and Bork, 2019). Average amino acid identity (AAI) between genomes was calculated with the ‘aai-matrix-diamond.sh’ script from the enveomics package (Rodriguez-R and Konstantinidis, 2016).

## Data availability

Raw DNA sequencing reads, cross-assembled metagenome sequences, MAG sequences and metadata from the Black Sea have been deposited at the European Nucleotide Archive as part of BioProject PRJNA649215.

## Detection of functional marker genes

Homologs of functional marker genes were detected with Hidden Markov Models (HMMs), in part obtained from the PFAM (El-Gebali et al., 2018) and TIGRFAM (Haft et al., 2012) databases (Table S4). HMMs for the detection of *dsrA*, *dsrB, dsrD*, *dsrE, dsrF, dsrH, dsrM, dsrK, dsrJ, dsrO, dsrP* and *dsrT* were kindly provided by Dr. Karthik Anantharaman (Anantharaman et al., 2018). The remaining HMMs^[[3]](#footnote-3)^ for detection of *dsrC*, *dsrL*, *fccB*/*soxF*, *fsr*, *mccA_sirA*, *phsA*/*psrA*/*sreA*, *rdlA*, *sat*, *soeA, sorA, sorT, soxL, sqrA*/*sqrD, sqrB*/*sqrC*, *sqrE*/*sqrF, tetH*, *tsdA* and *ttrA* were constructed with HMMER version 3.1b2 (Eddy, 2009) following selection of reliable, divergent protein sequences based on literature, SwissProt and InterPro family descriptions, multiple alignment with Clustal Omega (Sievers et al., 2011), and threshold evaluation with HMMER searches against the SwissProt and UniProtRefProt databases using the EBI HMMER web server (Finn et al., 2015). Because of the close homology of the alpha subunits of polysulfide reductase (*psrA*), thiosulfate reductase (*phsA*) and sulfur reductase (*sreA*), we constructed an HMM for the detection of this molybdoenzyme subfamily encompassing *phsA, psrA* and *sreA*. Notably, this subfamily includes *psrA*-like genes which are present in cultivated sulfur-oxidizing microorganisms. Similarly, the flavoprotein disulfide reductase family includes six different types of sulfide-quinone oxidoreductase (*sqrA-F*)(Gregersen et al., 2011) as well as flavocytochrome *c* sulfide dehydrogenase (known as *fccB*, *soxF* or *FCSD*)(Marcia et al., 2010). However, in contrast to *psrA-*like genes, the extensive functional and phylogenetic investigations into *sqr* and *fccB* genes (Han and Perner, 2015; Shuman and Hanson, 2016) allowed us to construct HMMs for the separate detection of *sqrA*/*sqrD*, *sqrE*/*sqrF* and *fccB*/*soxF*. For detection of *dsrD* in the metagenome assembly, the scaffolds were first six-frame translated into protein sequences with the *transeq* tool. Coding sequences of individual genomes were predicted with Prodigal (Hyatt et al., 2010). The translated metagenome scaffolds and genomic coding sequences were queried with the HMM profiles with *hmmsearch* using default parameter settings. Subsequently, scaffolds or coding sequences with hits that passed the bitscore cutoff (‘noise’ or ‘trusted’, depending on the profile) were extracted from the *hmmsearch* output using the Bio.SearchIO module of the Biopython package (Cock et al., 2009). Calculations were done within the Pandas package data analysis framework (McKinney, 2010).

**Table S4.** Publicly available HMM profiles used in this study.

| **Gene** | **PFAM/TIGRFAM** |
| --- | --- |
| *amo_mmoA* | TIGR03080 |
| *amo_mmoB* | TIGR03079 |
| *amo_mmoC* | TIGR03078 |
| *anfD* | TIGR01861 |
| *anfG* | TIGR02929 |
| *anfK* | TIGR02931 |
| *aprA* | TIGR02061 |
| *aprB* | TIGR02060 |
| *asrC* | TIGR02912 |
| Catalase_haem | PF00199 |
| Catalase_Mn | PF05067 |
| Catalase_peroxidase | TIGR00198 |
| *ccoN* | TIGR00780 |
| *ccoO* | TIGR00781 |
| *ccoP* | TIGR00782 |
| *ccoQ* | PF05545 |
| *coxA* | TIGR02891 |
| *coxB* | TIGR02866 |
| *cuyA* | TIGR01275 |
| *cydA* | PF01654 |
| *cydB* | PF02322 |
| *cyoA* | TIGR01433 |
| *cyoB* | TIGR02843 |
| *cyoC* | TIGR02842 |
| *cyoD* | TIGR02847 |
| *ddhA* | TIGR03479 |
| *dmsA* | TIGR02166 |
| *dmsC* | PF04976 |
| *mcrA* | TIGR03256 |
| *napA* | TIGR01706 |
| *napB* | PF03892 |
| *napC* | TIGR02161 |
| *narG_nxrA* | TIGR01580 |
| *narH* | TIGR01660 |
| *narI* | TIGR00351 |
| *nifD* | TIGR01282 |
| *nifH* | TIGR01287 |
| *nifK* | TIGR01286 |
| *nirB* | TIGR02374 |
| *nirD* | TIGR02378 |
| *nirK* | TIGR02376 |
| *nosZ* | TIGR04244 |
| *nosZ_2* | TIGR04246 |
| *nrfA* | PF02335 |
| *nrfB* | TIGR03146 |
| *nrfC* | TIGR03149 |
| *nrfD* | TIGR03148 |
| *nrfH* | TIGR03153 |
| *otr* | TIGR04315 |
| *qoxA* | TIGR01432 |
| *qoxB* | TIGR02882 |
| *qoxC* | TIGR02897 |
| *qoxD* | TIGR02901 |
| Rhodanese | PF00581 |
| *soxA* | TIGR04484 |
| *soxB* | TIGR04486 |
| *soxC* | TIGR04555 |
| *vnfD* | TIGR01860 |
| *vnfG* | TIGR02930 |
| *vnfK* | TIGR02932 |
| *xsc* | TIGR03457 |
| *soxX* | TIGR04485 |
| *soxY* | TIGR04488 |
| *soxZ* | TIGR04490 |

A phylogenetic reconstruction was made with the *dsrA* genes from all genomes from dysoxic marine waters, the Black Sea metagenome and reference *dsrA* genes from Anantharaman et al. (2018). To extract *dsrA* genes from the Black Sea metagenome, coding sequences were first predicted with Prodigal using the ‘-p meta’ option (Hyatt et al., 2010). HMM matches of less than 200 amino acids were discarded. The remaining sequences were aligned with Clustal Omega (Sievers et al., 2011). The alignment was curated by removing *dsrA* sequences with >70% gaps and dereplicating identical sequences. Subsequently, a bootstrapped phylogenetic tree was constructed with IQ-TREE v1.6.10 as described before using the LG+R4+F evolutionary model.

## Thermodynamic calculations

Gibbs free energies (ΔG [kJ/e^-^], Table S3) were calculated as follows. Gibbs free energies of formation from Thauer *et al.* (1977) were used to calculate the standard Gibbs free energies (ΔG^0^ [kJ/half-reaction]) of half-reactions. These ΔG^0^ values were corrected to a pH of 8 with the Nernst equation assuming an *in situ* temperature of 283 K. The concentrations of and ammonium, nitrite and nitrate in the ETSP OMZ core at 80 m depth were obtained from Canfield et al. (2010), and the concentrations of those compounds as well as sulfide in the Black Sea at 110 m depth were obtained from Sollai et al. (2019). Other concentrations were estimated (Table S6). Activities were calculated using activity coefficients from Millero and Schreiber (1982) estimated for 25‰ salinity and 298 K: ammonium, 0.64; monovalent anions, 0.58; divalent anions, 0.109; gases, 1.2. The activity of S^0^ in the Black Sea in the upper euxinic zone was previously estimated to be around 1 (Helz, 2014). For the ETSP OMZ core, we assumed S^0^ to be mostly colloidal elemental sulfur and thus used an activity of 1.2. The Gibbs free energies (ΔG [kJ/half-reaction]) were corrected for these activities, again with the Nernst equation, and divided by the number of electron per half-reaction to obtain the Gibbs free energy per electron (ΔG [kJ/e^-^]) for each half-reaction. The ΔG (kJ/e^-^) values for complete reactions were obtained by subtracting the ΔG (kJ/e^-^) of the electron donor half-reaction from that of the electron acceptor half-reaction (Table S3).

**Table S6.** Concentrations and partial pressures used for thermodynamic calculations. SCIs represent thiosulfate, tetrathionate and sulfite.

|  | Black Sea (110 m depth) | ETSP OMZ core station 3 (80 m depth) |
| --- | --- | --- |
| Acetate (M) | 1.00E-06 | 1.00E-06 |
| Ammonium (M) | 8.80E-06 | 1.0E-07 |
| Bicarbonate (M) | 3.0E-03 | 3.0E-03 |
| H_2_ (atm) | 1.0E-06 | 1.0E-06 |
| Methane (atm) | 1.0E-05 | 1.0E-05 |
| N_2_ (atm) | 0.8 | 0.8 |
| Nitrate (M) | 2.70E-08 | 1.2E-05 |
| Nitrite (M) | 1.30E-08 | 5.0E-06 |
| SCIs (except S^0^; M) | 1.00E-08 | 1.00E-08 |
| Sulfate (M) | 2.0E-02 | 2.0E-02 |
| Sulfide (M) | 4.60E-06 | 1.00E-08 |

# References

Anantharaman, K., Hausmann, B., Jungbluth, S.P., Kantor, R.S., Lavy, A., Warren, L.A. et al. (2018) Expanded diversity of microbial groups that shape the dissimilatory sulfur cycle. *ISME J* **12**: 1715-1728.

Bankevich, A., Nurk, S., Antipov, D., Gurevich, A.A., Dvorkin, M., Kulikov, A.S. et al. (2012) SPAdes: a new genome assembly algorithm and its applications to single-cell sequencing. *J Comput Biol* **19**: 455-477.

Boulegue, J., and Michard, G. (1979) Sulfur speciations and redox processes in reducing environments. In *Chemical Modeling in Aqueous Systems*: American Chemical Society Symposium Series 93, pp. 25-50.

Buchfink, B., Xie, C., and Huson, D.H. (2014) Fast and sensitive protein alignment using DIAMOND. *Nat Methods* **12**: 59.

Canfield, D.E., Stewart, F.J., Thamdrup, B., De Brabandere, L., Dalsgaard, T., Delong, E.F. et al. (2010) A cryptic sulfur cycle in oxygen-minimum-zone waters off the Chilean coast. *Science* **330**: 1375-1378.

Chaumeil, P.-A., Mussig, A.J., Hugenholtz, P., and Parks, D.H. (2019) GTDB-Tk: a toolkit to classify genomes with the Genome Taxonomy Database. *Bioinformatics* **36**: 1925-1927.

Cline, J.D. (1969) Spectrophotometric determination of hydrogen sulfide in natural waters. *Limnol Oceanogr* **14**: 454-458.

Cock, P.J., Antao, T., Chang, J.T., Chapman, B.A., Cox, C.J., Dalke, A. et al. (2009) Biopython: freely available Python tools for computational molecular biology and bioinformatics. *Bioinformatics* **25**: 1422-1423.

Dodt, M., Roehr, J.T., Ahmed, R., and Dieterich, C. (2012) FLEXBAR—flexible barcode and adapter processing for next-generation sequencing platforms. *Biology* **1**: 895-905.

Eddy, S.R. (2009) A new generation of homology search tools based on probabilistic inference. In *Genome Informatics 2009: Genome Informatics Series Vol 23*: World Scientific, pp. 205-211.

El-Gebali, S., Mistry, J., Bateman, A., Eddy, S.R., Luciani, A., Potter, S.C. et al. (2018) The Pfam protein families database in 2019. *Nucleic Acids Res* **47**: D427-D432.

Finn, R.D., Clements, J., Arndt, W., Miller, B.L., Wheeler, T.J., Schreiber, F. et al. (2015) HMMER web server: 2015 update. *Nucleic Acids Res* **43**: W30-38.

Finn, R.D., Coggill, P., Eberhardt, R.Y., Eddy, S.R., Mistry, J., Mitchell, A.L. et al. (2016) The Pfam protein families database: towards a more sustainable future. *Nucleic Acids Res* **44**: D279-285.

Ganesh, S., Parris, D.J., DeLong, E.F., and Stewart, F.J. (2014) Metagenomic analysis of size-fractionated picoplankton in a marine oxygen minimum zone. *ISME J* **8**: 187-211.

Gregersen, L.H., Bryant, D.A., and Frigaard, N.U. (2011) Mechanisms and evolution of oxidative sulfur metabolism in green sulfur bacteria. *Front Microbiol* **2**: 116.

Haft, D.H., Selengut, J.D., Richter, R.A., Harkins, D., Basu, M.K., and Beck, E. (2012) TIGRFAMs and Genome Properties in 2013. *Nucleic Acids Res* **41**: D387-D395.

Han, Y., and Perner, M. (2015) The globally widespread genus *Sulfurimonas*: versatile energy metabolisms and adaptations to redox clines. *Front Microbiol* **6**: 989.

Helz, G.R. (2014) Activity of zero-valent sulfur in sulfidic natural waters. *Geochem Trans* **15**: 13.

Hoang, D.T., Chernomor, O., von Haeseler, A., Minh, B.Q., and Vinh, L.S. (2017) UFBoot2: Improving the ultrafast bootstrap approximation. *Mol Biol Evol* **35**: 518-522.

Hyatt, D., Chen, G.-L., LoCascio, P.F., Land, M.L., Larimer, F.W., and Hauser, L.J. (2010) Prodigal: prokaryotic gene recognition and translation initiation site identification. *BMC Bioinformatics* **11**: 119.

Jørgensen, B.B., Fossing, H., Wirsen, C.O., and Jannasch, H.W. (1991) Sulfide oxidation in the anoxic Black Sea chemocline. *Deep Sea Research Part A Oceanographic Research Papers* **38**: S1083-S1103.

Kalyaanamoorthy, S., Minh, B.Q., Wong, T.K.F., von Haeseler, A., and Jermiin, L.S. (2017) ModelFinder: fast model selection for accurate phylogenetic estimates. *Nat Methods* **14**: 587.

Kang, D.D., Froula, J., Egan, R., and Wang, Z. (2015) MetaBAT, an efficient tool for accurately reconstructing single genomes from complex microbial communities. *PeerJ* **3**: e1165.

Letunic, I., and Bork, P. (2019) Interactive Tree Of Life (iTOL) v4: recent updates and new developments. *Nucleic Acids Res* **47**: W256-W259.

Li, H. (2013) Aligning sequence reads, clone sequences and assembly contigs with BWA-MEM. *arXiv preprint arXiv:13033997*.

Lüke, C., Speth, D.R., Kox, M.A.R., Villanueva, L., and Jetten, M.S.M. (2016) Metagenomic analysis of nitrogen and methane cycling in the Arabian Sea oxygen minimum zone. *PeerJ* **4**: e1924.

Marcia, M., Ermler, U., Peng, G., and Michel, H. (2010) A new structure-based classification of sulfide:quinone oxidoreductases. *Proteins* **78**: 1073-1083.

McKinney, W. (2010) Data structures for statistical computing in python. In *Proceedings of the 9th Python in Science Conference*: SciPy Austin, TX, pp. 51-56.

Mendler, K., Chen, H., Parks, D.H., Lobb, B., Hug, L.A., and Doxey, A.C. (2019) AnnoTree: visualization and exploration of a functionally annotated microbial tree of life. *Nucleic Acids Res* **47**: 4442-4448.

Millero, F.J., and Schreiber, D.R. (1982) Use of the ion-pairing model to estimate activity-coefficients of the eonic components of natural waters. *Am J Sci* **282**: 1508-1540.

Nguyen, L.T., Schmidt, H.A., von Haeseler, A., and Minh, B.Q. (2015) IQ-TREE: a fast and effective stochastic algorithm for estimating maximum-likelihood phylogenies. *Mol Biol Evol* **32**: 268-274.

Parks, D.H., Imelfort, M., Skennerton, C.T., Hugenholtz, P., and Tyson, G.W. (2015) CheckM: assessing the quality of microbial genomes recovered from isolates, single cells, and metagenomes. *Genome Res* **25**: 1043-1055.

Parks, D.H., Rinke, C., Chuvochina, M., Chaumeil, P.-A., Woodcroft, B.J., Evans, P.N. et al. (2017) Recovery of nearly 8,000 metagenome-assembled genomes substantially expands the tree of life. *Nat Microbiol* **2**: 1533-1542.

Rodriguez-R, L.M., and Konstantinidis, K.T. (2016) The enveomics collection: a toolbox for specialized analyses of microbial genomes and metagenomes. *PeerJ Prepr* **4**: e1900v1901.

Shuman, K.E., and Hanson, T.E. (2016) A sulfide:quinone oxidoreductase from *Chlorobaculum tepidum* displays unusual kinetic properties. *FEMS Microbiol Lett* **363**.

Sievers, F., Wilm, A., Dineen, D., Gibson, T.J., Karplus, K., Li, W. et al. (2011) Fast, scalable generation of high‐quality protein multiple sequence alignments using Clustal Omega. *Mol Syst Biol* **7**: 539.

Sollai, M., Villanueva, L., Hopmans, E.C., Reichart, G.J., and Sinninghe Damste, J.S. (2019) A combined lipidomic and 16S rRNA gene amplicon sequencing approach reveals archaeal sources of intact polar lipids in the stratified Black Sea water column. *Geobiology* **17**: 91-109.

Tully, B.J., Graham, E.D., and Heidelberg, J.F. (2018) The reconstruction of 2,631 draft metagenome-assembled genomes from the global oceans. *Sci Data* **5**: 170203.

Villanueva, L., von Meijenfeldt, F.A.B., Westbye, A.B., Yadav, S., Hopmans, E.C., Dutilh, B.E., and Sinninghe Damste, J.S. (2020) Bridging the membrane lipid divide: bacteria of the FCB group superphylum have the potential to synthesize archaeal ether lipids. *Environ Microbiol*. (in press)

von Meijenfeldt, F.A.B., Arkhipova, K., Cambuy, D.D., Coutinho, F.H., and Dutilh, B.E. (2019) Robust taxonomic classification of uncharted microbial sequences and bins with CAT and BAT. *bioRxiv*: 530188.

1. https://www.bioinformatics.babraham.ac.uk/projects/fastqc/ [↑](#footnote-ref-1)
2. https://github.com/rambaut/figtree/releases [↑](#footnote-ref-2)
3. https://github.com/dmvvliet/protein-HMMs [↑](#footnote-ref-3)
