# Supplementary material for: The bacterial sulfur cycle in expanding dysoxic and euxinic marine waters
Source: Environ Microbiol. 2020 Oct 18;23(6):2834–57. doi: 10.1111/1462-2920.15265 (PMC8359478; doi:10.1111/1462-2920.15265)
Supplement: Supplementary file 6 — Appendix S2. Supplementary Information Protologue. [file EMI-23-2834-s006.docx]

**Description of novel taxa proposed in this study**

For uncultured taxa, high-quality metagenome-assembled genomes (MAGs) serve as type material as outlined by Konstantinidis et al. (2017) and Chuvochina et al. (2019). MAG sequences were deposited at the European Nucleotide Archive as part of BioProject PRJNA649215.

**Description of *^U^Sulfurimonas ponti* sp. nov.**

*^U^Sulfurimonas ponti* (pon’ti. L. gen. masc. n. *ponti*, of the sea). Genome analysis predicts the capacity for dissimilatory sulfide oxidation, nitrate reduction, ammonification and oxygen-based respiration. Appears to be a marine chemolithoautotrophic sulfur-oxidizing bacterium preferring euxinic waters, based on its distribution in the partially euxinic Black Sea water column. The type material is genome assembly NIOZ-UU79^T^ with accession number JACNLH000000000.

**Description of *^U^Thiopontia* gen. nov., sp. nov.**

*^U^Thiopontia* (Thi.o.pon’tia. Gr. neut. n. *theion/θεῖον*, sulfur; Gr. fem. adj. *pontia/ποντιά*, from the sea; N.L. fem. n. *Thiopontia*, sulfur oxidizer from the sea). Member of the class *Gammaproteobacteria*. Type species is *^U^Thiopontia autotrophica*.

**Description of *^U^Thiopontia autotrophica* sp. nov.**

*^U^Thiopontia autotrophica* (au.to.tro’phi.ca. Gr. masc. pron. *autos*/*αὑτός*, self; Gr. masc. adj. *trophikos*/*τροφικός*, one who feeds; N.L. fem. adj. *autotrophica*, one who feeds herself, autotrophic). Genome analysis predicts the capacity for dissimilatory oxidation of sulfide, elemental sulfur and dimethylsulfide, complete denitrification, oxygen-based respiration, and autotrophy. Appears to be a marine chemolithoautotrophic sulfur-oxidizing bacterium, based on its distribution in the partially euxinic Black Sea water column. The type material is genome assembly NIOZ-UU100^T^ with accession number JACNFK000000000.

**Description of ‘*Candidatus* Pseudothioglobus’ gen. nov.**

*^U^Pseudothioglobus* (Pseu.do.thi.o.glo’bus. Gr. masc. adv. *pseudos*/*ψεῦδος*, false; Gr. neut. n. *theion/θεῖον*, sulfur; L. masc. n. *globus*, ball, sphere; N.L. masc. n. *Pseudothioglobus*, false sulfur-oxidizing sphere). Member of the class *Gammaproteobacteria*. Type species is ‘Candidatus *Pseudothioglobus singularis’* (Marshall and Morris, 2013) comb. nov.

**Description of ‘*Candidatus* Pseudothioglobus singularis’ comb. nov.**

*Candidatus* ‘Pseudothioglobus singularis’ (basionym, ‘*Candidatus* Thioglobus singularis’ Marshall and Morris 2013; sin.gu.la’ris. L. adj. *singularis*, alone, singular). Type material is strain PS1^T^.

**Description of *^U^Pseudothioglobus aerophilus* sp. nov.**

*^U^Pseudothioglobus aerophilus* (ae.ro.phi’lus. Gr. n. *aer*/*ἀήρ*, air; Gr. masc. adj. *philos*/*φίλος*, loving; N.L. masc. adj. *aerophilus* air-loving, referring to a strict preference for oxygenated water). Appears to be a strictly aerobic marine bacterium, based on its distribution in the partially euxinic Black Sea water column. The type material is genome assembly NIOZ-UU104^T^ with accession number JACNFO000000000.

**Emended description of the genus ‘*Candidatus* Thioglobus’ (Marshall and Morris, 2013)**

Member of the class *Gammaproteobaceria*. The type species is ‘*Candidatus* Thioglobus autotrophicus’ (Shah et al., 2017).

**Description of *^U^Thioglobus pontius* sp. nov.**

*^U^Thioglobus pontius* (pon’ti.us. Gr. masc. adj. *pontios*/*πόντιος*, from the sea; N.L. masc. adj. *pontius*, from the sea). Genome analysis predicts the capacity for dissimilatory oxidation of sulfide, elemental sulfur, thiosulfate and dimethylsulfide, incomplete denitrification of nitrate to nitric/nitrous oxide and microaerobic respiration. Appears to be a marine chemolithoautotrophic sulfur-oxidizing bacterium, based on its distribution in the partially euxinic Black Sea water column. The type material is genome assembly NIOZ-UU116^T^ with accession number JACNGB000000000.

**Description of *^U^Desulfatifera* gen. nov.**

*^U^Desulfatifera* (De.sul.fa.ti.fe’ra. L. pref. *de,* off; N.L. masc. n. *sulfas –atis,* sulfate; L. fem. n. *fera*, a wild beast; N.L. fem. n. *Desulfatifera,* a wild sulfate reducer). Member of the family *Desulfobulbaceae*. Type species is *^U^Desulfatifera sulfidica*.

**Description of *^U^Desulfatifera sulfidica* sp. nov.**

*^U^Desulfatifera sulfidica* (sul.fi’di.ca. N.L. fem. adj. *sulfidica*, sulfidic, referring to a high relative abundance in the sulfidic part of the Black Sea). Genome analysis predicts the capacity for the dissimilatory reduction of sulfate and tetrathionate, the ammonification of nitrite, and potentially diazotrophy. Appears to be have a preference for euxinic marine waters, based on its distribution in the partially euxinic Black Sea water column. The type material is genome assembly NIOZ-UU81^T^ with accession number JACNLK000000000.

**Description of *^U^Desulfobia* gen. nov.**

*^U^Desulfobia* (De.sul.fo.bi’a. L. pref. *de,* off; L. neut. n. *sulfur,* brimstone, sulfur; Gr. masc. n. *bios*/*βίος*, life; N.L. fem. n. *Desulfobia,* a sulfur-reducing life-form). Member of the family *Desulfobulbaceae*. Type species is *^U^Desulfobia pelagia*.

**Description of *^U^Desulfobia pelagia* sp. nov.**

*^U^Desulfobia pelagia* (pe.la’gi.a. Gr. fem. adj. *pelagia*/*πελάγια*, from the sea). Genome analysis predicts the capacity for the dissimilatory reduction of sulfate, tetrathionate, the ammonification of nitrite, and diazotrophy. May reduce and/or disproportionate elemental sulfur and/or thiosulfate, based on its genetic capacity and distribution in the partially euxinic Black Sea water column. The type material is genome assembly NIOZ-UU47^T^ with accession number JACNJZ000000000.

**Description of *^U^Desulfacyla* gen. nov.**

*^U^Desulfacyla* (De.sul.fa’cy.la. L. pref. *de,* off; N.L. neut. n. *sulfur,* brimstone, sulfur; L. fem. n. *akylos*/*ἄκυλος*, acorn; N.L. fem. n. *Desulfacylus,* a sulfur-reducing acorn). Member of the class *Deltaproteobacteria*, sister group of genus *Desulfatiglans*. Type species is *^U^Desulfacyla euxinica*.

**Description of *^U^Desulfacyla euxinica* sp. nov.**

*^U^Desulfacyla euxinica* (eu.xi’ni.ca. Gr. masc. adj. *euxeinos*/*εὔξεινος*, hospitable; N.L. fem. adj. *euxinica*, euxinic, referring to a high relative abundance in the euxinic part of the Black Sea). Genome analysis predicts the capacity for the dissimilatory reduction of tetrathionate, dimethylsulfoxide, and possibly elemental sulfur, thiosulfate and nitrate. Thrives in both suboxic and euxinic marine waters, based on its distribution in the Black Sea water column. The type material is genome assembly NIOZ-UU27^T^ with accession number JACNJD000000000.

**Description of *^U^Desulfobacula maris* sp. nov.**

*^U^Desulfobacula maris* (ma’ris. L. gen. neut. n. *maris*, of the sea). Genome analysis predicts the capacity for the dissimilatory reduction of sulfate, tetrathionate, dimethylsulfoxide, and possibly elemental sulfur, thiosulfate, nitrate, and oxygen. May oxidize sulfide and disproportionate elemental sulfur and/or thiosulfate, based on its genetic capacity and distribution in the Black Sea water column. The type material is genome assembly NIOZ-UU16^T^ with accession number JACNHV000000000.

**Description of *^U^Desulfatibia* gen. nov.**

*^U^Desulfatibia* (De.sul.fa.ti.bi’a. L. pref. *de,* off; N.L. masc. n. *sulfas –atis,* sulfate; Gr. masc. n. *bios*/*βίος*, life; N.L. fem. n. *Desulfatibia,* a sulfate-reducing life-form). Member of the order *Desulfobacterales*. Type species is *^U^Desulfatibia profunda*.

**Description of *^U^Desulfatibia profunda* sp. nov.**

*^U^Desulfatibia profunda* (pro.fun’da. L. fem. adj. *profunda,* deep, referring to a high relative abundance in the deep, euxinic part of the Black Sea). Appears to be a sulfate-reducing bacterium with a strictly euxinic habitat, based on its genetic capacity and occurrence in exclusively the euxinic part of the Black Sea. The type material is genome assembly NIOZ-UU30^T^ with accession number JACNJH000000000.

**Description of *^U^Desulfatibia vada* sp. nov.**

*^U^Desulfatibia vada* (va’da. L. fem. adj. *vada,* shallow, referring to a high relative abundance in the suboxic zone of the Black Sea, which is more shallow than the euxinic zone). Genome analysis predicts the capacity for the reduction of sulfate, tetrathionate, and possibly nitrate (to nitrite or ammonia) and oxygen. May oxidize sulfide and/or disproportionate elemental sulfur, based on its genetic capacity and occurrence in exclusively the euxinic part of the Black Sea. The type material is genome assembly NIOZ-UU17^T^ with accession number JACNIG000000000.

**Description of *^U^Desulfaltia* gen. nov.**

*^U^Desulfaltia* (De.sul.fal’ti.a. L. pref. *de,* off; L. neut. n. *sulfur,* brimstone, sulfur; L. masc. adj. *altus*, deep; N.L. fem. n. *Desulfaltia,* a sulfate reducer from the deep). Member of the order *Desulfobacterales*. Type species is *^U^Desulfaltia bathyphila*.

**Description of *^U^Desulfaltia bathyphila* sp. nov.**

*^U^Desulfaltia bathyphila* (ba.thy.phi’la. Gr. masc. adj. *bathys*/*βαθύς*, deep; Gr. masc. adj. *philos*/*φίλος*, loving; N.L. fem. adj. *bathyphila*, deep-loving, referring to a high relative abundance in the deep, euxinic part of the Black Sea). Appears to be a sulfate-reducing bacterium with a strictly euxinic habitat, based on its genetic capacity and occurrence in exclusively the euxinic part of the Black Sea. The type material is genome assembly NIOZ-UU82^T^ with accession number JACNLL000000000.

**Description of *^U^Desulfolinea* gen. nov.**

*^U^Desulfolinea* (De.sul.fo.li’ne.a. L. pref. *de,* off; L. neut. n. *sulfur,* brimstone, sulfur; L. fem. n. *linea*, line, string; N.L. fem. n. *Desulfolinea,* a sulfur-reducing string or line). Member of the order *Anaerolineales*. Type species is *^U^Desulfolinea nitratireducens*.

**Description of *^U^Desulfolinea nitratireducens* sp. nov.**

*^U^Desulfolinea nitratireducens* (ni.tra.ti.re.du’cens. N.L. masc. n. *nitras*, -*atis*, nitrate; L. v. *reducere*, -*o*, to bring back; N.L. part. adj. *nitratireducens*, nitrate-reducing). Genome analysis predicts the capacity for the reduction of sulfite, tetrathionate and nitrate, and the oxidation of dimethylsulfide. May oxidize sulfide, ammonify nitrite and respire oxygen microaerobically, based on its genetic capacity and its high relative abundance in the suboxic zone of the Black Sea. The type material is genome assembly NIOZ-UU36^T^ with accession number JACNJN000000000.

**References**

Chuvochina, M., Rinke, C., Parks, D.H., Rappé, M.S., Tyson, G.W., Yilmaz, P. et al. (2019) The importance of designating type material for uncultured taxa. *Systematic and Applied Microbiology* **42**: 15-21.

Konstantinidis, K.T., Rosselló-Móra, R., and Amann, R. (2017) Uncultivated microbes in need of their own taxonomy. *ISME J* **11**: 2399-2406.

Marshall, K.T., and Morris, R.M. (2013) Isolation of an aerobic sulfur oxidizer from the SUP05/Arctic96BD-19 clade. *ISME J* **7**: 452-455.

Shah, V., Chang, B.X., and Morris, R.M. (2017) Cultivation of a chemoautotroph from the SUP05 clade of marine bacteria that produces nitrite and consumes ammonium. *ISME J* **11**: 263–271.
